# The roles of epigenetic regulators in plant regeneration: Exploring patterns amidst complex conditions

**DOI:** 10.1093/plphys/kiae042

**Published:** 2024-01-30

**Authors:** Jiawen Li, Qiyan Zhang, Zejia Wang, Qikun Liu

**Affiliations:** State Key Laboratory of Protein and Plant Gene Research, School of Advanced Agricultural Sciences, Peking University, Beijing 100871, China; State Key Laboratory of Protein and Plant Gene Research, School of Advanced Agricultural Sciences, Peking University, Beijing 100871, China; State Key Laboratory of Protein and Plant Gene Research, School of Advanced Agricultural Sciences, Peking University, Beijing 100871, China; State Key Laboratory of Protein and Plant Gene Research, School of Advanced Agricultural Sciences, Peking University, Beijing 100871, China

## Abstract

Plants possess remarkable capability to regenerate upon tissue damage or optimal environmental stimuli. This ability not only serves as a crucial strategy for immobile plants to survive through harsh environments, but also made numerous modern plant improvements techniques possible. At the cellular level, this biological process involves dynamic changes in gene expression that redirect cell fate transitions. It is increasingly recognized that chromatin epigenetic modifications, both activating and repressive, intricately interact to regulate this process. Moreover, the outcomes of epigenetic regulation on regeneration are influenced by factors such as the differences in regenerative plant species and donor tissue types, as well as the concentration and timing of hormone treatments. In this review, we focus on several well-characterized epigenetic modifications and their regulatory roles in the expression of widely studied morphogenic regulators, aiming to enhance our understanding of the mechanisms by which epigenetic modifications govern plant regeneration.

## Teaser

Epigenetic modifications change during plant regeneration and play complex roles in this process.

## Introduction

Plants possess a remarkable capacity for regeneration to repair their damaged tissues. This feature has become an agronomically important trait, serving as the foundation for key plant improvement procedures, such as grafting, haplotype breeding, and genetic transformation. Plants can regenerate via a diverse range of developmental processes (Box 1). At the cellular level, regardless of the type of plant regeneration, a common key feature is the acquisition of cell pluripotency through a transition in cell fate from terminally differentiated somatic cells. This process is associated with an intensive reprogramming of gene expression profiles but involves limited, if any, changes in genetic information. Instead, dynamic epigenetic changes, both globally and in key developmental genes, are believed to be critical for this process.

Box 1. **Different modes of plant regeneration**

- Somatic embryogenesis: A single somatic cell can be induced by auxin to form an embryo, which develops into a whole plant. Somatic embryos can either be generated directly from individual somatic cells or indirectly from embryonic callus.
- De novo organogenesis: This includes de novo shoot organogenesis and de novo root organogenesis. In the direct de novo organogenesis pathway, shoots or roots are directly produced from somatic tissues and organs. In the indirect pathway, pluripotent callus is produced from detached plant explants, from which shoots and roots are subsequently generated.

In this review, we summarize advances in studying some of the most well-characterized epigenetic modifications during plant regeneration, including DNA methylation, trimethylation at lysine 27 of histone H3 (H3K27me3), trimethylation at lysine 4 of histone H3 (H3K4me3), and histone H3/H4 acetylation (H3/H4 acetylation). We then focus on key morphogenetic gene families to summarize and illustrate the possible mechanisms of epigenetic regulation associated with plant regeneration. For additional details, we recommend excellent recent reviews on this topic (Lee and Seo 2018; Aflaki et al. 2022; Chen et al. 2023b; Liu et al. 2023b).

## DNA methylation and plant regeneration

DNA methylation in plants occurs in 3 primary sequence contexts, CG, CHG, and CHH (where H represents A, T, or C), which are catalyzed by different families of DNA methyltransferases. In Arabidopsis (*Arabidopsis thaliana*), METHYLTRANSFERASE 1 (MET1) maintains CG DNA methylation during DNA replication, while CHROMOMETHYLASE 3 (CMT3) is primarily responsible for CHG DNA methylation. CHH DNA methylation is either de novo deposited in euchromatic regions by the RNA-directed DNA METHYLATION (RdDM) pathway enzyme DOMAIN REARRANGED METHYLTRANSFERASE 2 (DRM2) or maintained in heterochromatic regions by CMT2 (Law and Jacobsen 2009; Matzke and Mosher 2014). Additionally, DNA methylation in all sequence contexts can be actively removed by 5-methylcytosine DNA glycosylases, including REPRESSOR OF SILENCING 1 (ROS1) and its homologous proteins TRANSCRIPTIONAL ACTIVATOR DEMETER (DME), DEMETER-LIKE 2 (DML2), and DML3 (Zhang et al. 2018).

### Variation in DNA methylation and regeneration efficiency

Dynamic changes in global DNA methylation levels and local distribution patterns are recognized as common features of plant regeneration in various plant species (Chakrabarty et al. 2003; Tanurdzic et al. 2008; Stroud et al. 2013; Zakrzewski et al. 2017; Grzybkowska et al. 2018). The specific patterns of DNA methylation can be influenced by several factors, including genetic variation, the source of the explant, and experimental conditions (Loschiavo et al. 1989; Santos and Fevereiro 2002; Chakrabarty et al. 2003; Tanurdzic et al. 2008; Shibukawa et al. 2009; Stroud et al. 2013; Gao et al. 2014; Zakrzewski et al. 2017; Grzybkowska et al. 2018). For example, genotype-dependent DNA methylomes have been observed in different cultivars, such as japonica vs. indica rice (*Oryza sativa*) and Lumian vs. Yuzao cotton (*Gossypium hirsutum*), which are also associated with differential regenerative capabilities (Abe and Futsuhara 1986; Altpeter et al. 2016; Hsu et al. 2018; Guo et al. 2020). Mechanistically, since DNA methylation occurs in various sequence contexts (CG, CHG, and CHH) and is catalyzed by distinct families of DNA methyltransferases (Wendte and Schmitz 2018), changes in DNA sequences can directly affect the target recognition of these enzymes. Alternatively, genetic variations in genes encoding DNA methyltransferases or demethylases may lead to differences in gene expression patterns and altered functions of these enzymes, with pleiotropic effects on the DNA methylome. Similarly, developmental stage-dependent changes in DNA methylation and plant regeneration efficiency have also been reported in various plant organs and species (Becerra et al. 2004; Cardoza and Stewart 2004; Naing et al. 2015; Zhang et al. 2018; Sun et al. 2020; Tang et al. 2020).

**Advances**

- Time-scale profiling using single-cell sequencing techniques has begun to offer cellular-level details of cell type composition and cell fate transitions during plant regeneration.
- Multiomics profiling techniques offer a genome-wide perspective on the transition of chromatin states and gene expression patterns that underlie changes in plant cell fate.
- Central elements in various plant signaling pathways, including TOR kinase and PIF transcription factors, directly modify or interact with epigenetic complexes, such as PRC2 and INO80, to regulate plant development and organogenesis.

However, interpreting the causal relationship between DNA methylation and plant regeneration efficiency is a complex matter. First, at a global scale, a linear relationship between changes in DNA methylation and gene expression is often lacking (Berdasco et al. 2008; Stroud et al. 2013; Guo et al. 2020; Sun et al. 2020; Liu et al. 2022; Zheng et al. 2022). Second, DNA methylation undergoes dynamic changes during plant regeneration. For instance, during cotton regeneration, there is a decrease in CHH methylation at the embryonic calli stage but not at the nonembryonic calli stage (Guo et al. 2020). Similarly, the total level of DNA methylation in rapeseed (*Brassica napus*) calli appears to fluctuate across different stages of regeneration, displaying 2 peaks at Day 12 and Day 30 on callus induction medium (CIM; Gao et al. 2014).

### DNA methylation and somaclonal variation

It has long been observed that plants regenerated through tissue culture often exhibit diverse phenotypic traits that are distinct from each other and from the donor plants. This phenomenon is known as somaclonal variation (Bairu et al. 2010). Similarly, only a limited number of loci showed common changes in DNA methylation in different rice regenerants (Stroud et al. 2013). One possible underlying mechanism involves stochastic changes in the genetic material of somatic cells due to random transposable element insertions (Ozeki et al. 1997; Ong-Abdullah et al. 2015). These changes may also be attributed to the dysregulated functions of DNA methyltransferases and the genome-wide perturbation of DNA methylation (Tanurdzic et al. 2008). Supporting this notion, research has confirmed that all major Arabidopsis methyltransferase and demethylase genes, including *MET1*, *CMT2*, *CMT3*, *DRM1*, and *ROS1*, undergo dynamic changes in expression during callus proliferation (Grzybkowska et al. 2018; Liu et al. 2018a, 2022; Sun et al. 2020).

The stochastic changes in DNA methylation may also occur at both the cellular and locus levels. DNA methylation patterns have been profiled in plants using maize microspores at single-cell resolution through bisulfite-converted randomly integrated fragments sequencing (BRIF-Seq) and using soybean single-cell root hairs (Hossain et al. 2017; Li et al. 2019). Alternative methods for profiling cell type–specific epigenetic information have also been reported, such as fluorescence-activated cell sorting (FACS) coupled with whole-genome bisulfite sequencing (WGBS) or Chromatin Immunoprecipitation sequencing (Kawakatsu et al. 2016; Lee et al. 2019b; Zhu et al. 2023) and single-cell Assay for Transposase-Accessible Chromatin with sequencing (Marand et al. 2021a, 2021b). However, despite these advances, investigating DNA methylation at single-cell resolution in the context of plant regeneration has posed technical challenges.

Analysis at the whole-genome scale has uncovered distinct patterns of changes in DNA methylation in various genomic features during plant regeneration (Tanurdzic et al. 2008; Stroud et al. 2013; Shim et al. 2022). For instance, during the transition from leaves to calli in Arabidopsis, changes in DNA methylation predominantly occur in transposable element regions, sparing genic regions (Shim et al. 2022). Additionally, dedifferentiated Arabidopsis suspension cells exhibit hyper-DNA methylation in euchromatin and hypo-DNA methylation in heterochromatin (Tanurdzic et al. 2008). In regenerated rice plants, the loss of DNA methylation is enriched in gene promoters (Stroud et al. 2013). As different plant species possess varying genomic compositions, including differences in the types and enrichment of repetitive sequences (IWGSC 2018; Shang et al. 2023; Chen et al. 2023a), the response of DNA methylation at the genome-wide scale may significantly differ across these diverse plant species.

## Characteristics and roles of H3K27me3 in plant regeneration

### Effects of H3K27me3 deposition

POLYCOMB REPRESSIVE COMPLEX 2 (PRC2) plays pivotal roles in controlling both animal and plant development through the deposition of H3K27me3 histone modifications (Whitcomb et al. 2007; Bieluszewski et al. 2021). In Arabidopsis, PRC2 encompasses 3 homologs of H3K27me3 methyltransferases: CURLY LEAF (CLF), SWINGER (SWN), and MEDEA (MEA); 3 homologs of PRC2 scaffold proteins: EMBRYONIC FLOWER2 (EMF2), VERNALIZATION 2 (VRN2), and FERTILIZATION-INDEPENDENT SEED 2 (FIS2); the H3K27me3-binding subunit FERTILIZATION-INDEPENDENT ENDOSPERM (FIE); and the WD-40 domain protein MULTICOPY SUPPRESSOR OF IRA (MSI1; Bieluszewski et al. 2021).

A deficiency in PRC2 function leads to irregular organ development and homeotic transformations in plants, such as changes from hypocotyls and roots to flower-like organs (Goodrich et al. 1997; Kinoshita et al. 2001; Holec and Berger 2012). The disrupted maintenance of cell fate can be attributed to disturbances in the expression patterns of cell identity genes that are normally maintained by PRC2-mediated H3K27me3 deposition. The critical role of PRC2 in regulating cell fate transitions is further underscored by the substantially disrupted progress of plant regeneration.

Numerous lines of evidence support the crucial function of PRC2 in preserving cell identities in a differentiated state and its overall inhibitory effects on callus formation and somatic embryogenesis (Mozgová et al. 2017). For instance, an analysis using Arabidopsis mutants with impaired PRC2 function demonstrated that the terminally differentiated root hair cells undergo additional rounds of mitotic divisions, leading to the development of callus-like tissue, which can eventually give rise to somatic embryos in these mutants (Ikeuchi et al. 2015). Moreover, the expression of a nonmodifiable histone variant, H3 at residue K27 with an alanine (H3.3^K27A^), in Arabidopsis resulted in enhanced callus formation (Fal et al. 2023). Similarly, in response to wounding, the expression of H3.15, an Arabidopsis H3 variant lacking lysine residue 27, is induced. This induction promotes cell proliferation and callus formation by reducing the levels of H3K27me3 (Yan et al. 2020). The presence and sequence conservation of H3.15 in multiple eudicots species suggest that the wounding-induced incorporation of H3.15 and its promotion of callus formation may serve as a conserved wounding response mechanism in plants (Yan et al. 2020).

In addition to genetic evidence, the use of GSK-J4, a small molecule that hinders the activity of H3K27me3 demethylases, markedly diminished callus development in peach (*Prunus persica*) leaf explants (Zheng et al. 2022). Taken together, all of the studies support the idea that decreased H3K27me3 levels facilitate the initial phases of plant regeneration, where cells undergo dedifferentiation and proliferate to attain pluripotency.

While deficient PRC2 function can lead to the spontaneous formation of callus from root hair cells (Chanvivattana et al. 2004; Ikeuchi et al. 2015), Arabidopsis *clf-50 swn-1* mutants lost the ability to generate callus from leaf blades on CIM (He et al. 2012). This implies that other factors also influence plant regeneration in PRC2-deficient mutants. Notably, callus induced from leaf blades lacks the embryonic features observed in callus spontaneously derived from root hairs and cotyledons, as evidenced by the absence of lipid accumulation (He et al. 2012; Ikeuchi et al. 2015). Therefore, the size and proliferation rate of callus do not necessarily reflect the efficiency of PRC2 in establishing cell pluripotency. In support of this notion, even though the incorporation of the H3.3^K27A^ histone variant improved callus production, these calli encountered difficulties in generating functional shoots (Fal et al. 2023).

As mentioned above, in mutants defective in PRC2 function, the seemingly contradictory observation of spontaneous callus formation from root hairs and impaired callus formation in leaf blades may also be related to the specific mutant alleles used. These studies have uncovered the involvement of different alleles and genetic backgrounds, including hypomorphic alleles such as *swn-1* in the Ws background (He et al. 2012) and *swn-7* in the Col-0 background (Ikeuchi et al. 2015), as well as the null *swn-3* allele (Chanvivattana et al. 2004). Additionally, callus formation closely mirrors the developmental process of lateral roots (Sugimoto et al. 2010). Therefore, the transition mode of cell fate must differ when using different types of explant tissues, such as root segments, cotyledons, and leaf blades. At the cellular level, this necessitates distinct reprogramming patterns mediated by PRC2, resembling the creation of “newspaper blackout poems,” as recently proposed (Bieluszewski et al. 2021).

### Effects of active H3K27me3 removal

In both animals and plants, methylation at histone tails can be actively removed by Jumonji C (JmjC) domain proteins (Crevillén 2020). The Arabidopsis genome harbors 21 JmjC family genes (Lu et al. 2008), among which 5 family members, including *JUMONJI 13* (*JMJ13*), *JMJ30*, *JMJ32*, *EARLY FLOWERING 6* (*ELF6*/*JMJ11*), and *RELATIVE OF EARLY FLOWERING 6* (*REF6*/*JMJ12*), exhibit substrate specificity toward H3K27me3 and play roles in regulating plant development (Lu et al. 2008, 2011; Crevillén et al. 2014; Gan et al. 2014; Zheng et al. 2019; Crevillén 2020).

Upon wounding, the immediate activation of many genes that regulate callus formation was observed, such as *WOUND-INDUCED DEDIFFERENTIATION 3* (*WIND3*), *ETHYLENE RESPONSE FACTOR 115* (*ERF115*), *PLETHORA 3* (*PLT3*), *PLT4*, and *PLT5* (Ikeuchi et al. 2017). Since a number of these genes were previously silenced and marked with H3K27me3 before wounding, their rapid activation upon wounding indicates that the active demethylation of H3K27me3 is required (Ikeuchi et al. 2017). Supporting this concept, JMJ30 was demonstrated to promote callus proliferation by binding to and activating a subset of *LATERAL ORGAN BOUNDARIES DOMAIN* (*LBD*) genes (Lee et al. 2018b). Interestingly, in this context, JMJ30 was found to primarily demethylate trimethylation at lysine 9 of histone H3 (H3K9me3) at *LBD16* and *LBD29*, rather than H3K27me3 (Lee et al. 2018b). Therefore, further research is needed to determine whether JMJ30-mediated H3K27me3 demethylation also contributes to its role in regulating plant regeneration. Alternatively, JMJ30 may exhibit distinct substrate specificity in a locus- or cell type–dependent manner, as recently shown for JMJ16 (Wang et al. 2020b).

Apart from JMJ30, the roles of other H3K27me3 demethylases in regulating plant regeneration remain largely uncharacterized. Indirect evidence suggests that these additional H3K27me3 demethylases may also play a part in regulating plant regeneration. For instance, *CUP-SHAPED COTYLEDON 1* (*CUC1*), a gene with a crucial role in regulating shoot formation, has been identified as a target of REF6 (Daimon et al. 2003; Cui et al. 2016; Cao et al. 2023). In rice, WUSCHEL-RELATED HOMEOBOX 11 (WOX11) recruits the H3K27me3 demethylase JMJ705 to genes involved in shoot development, resulting in reduced H3K27me3 accumulation and increased gene expression (Cheng et al. 2018). Therefore, further research is necessary to gain a comprehensive understanding of the roles of other H3K27me3 demethylases in plant regeneration.

### Passive dilution of H3K27me3 during plant regeneration

In addition to active H3K27me3 demethylation, the passive dilution of H3K27me3 marks through mitotic cell division may also contribute to H3K27me3 reprogramming during plant regeneration (Hugues et al. 2020; Stewart-Morgan et al. 2020). The early phase of plant regeneration is characterized by rapid cell division and the upregulation of many cell division marker genes, including *CYCB1;1*, *CYCLIN D3;1-3* (*CYCD3;1-3*), and *E2F TRANSCRIPTION FACTOR 3a* (*E2Fa*; He et al. 2012; Ikeuchi et al. 2017; Liu et al. 2018a). Treatment with olomoucine, a cyclin-dependent kinase inhibitor, not only inhibited cell cycle progression but also delayed the reduction of H3K27me3 at the *WUSCHEL* (*WUS*) locus and the induction of *WUS* expression (Zhang et al. 2017).

**Figure 1.**
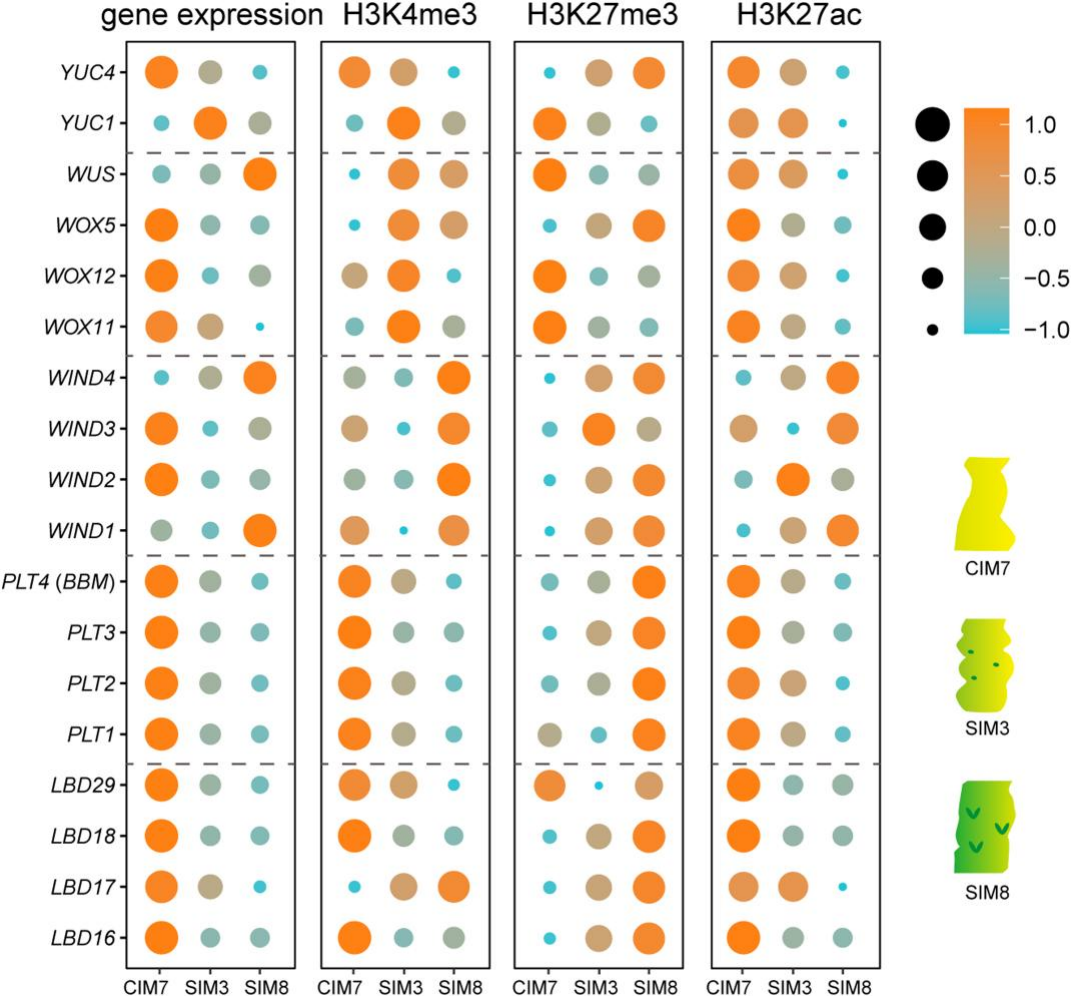
Dot plots depicting the expression levels of Arabidopsis genes and the density of H3K4me3, H3K27me3, and H3K27ac within 1.5 kb upstream and downstream of the genes. The size of the dots represents the absolute expression level of the genes or the level of each epigenetic modification on corresponding gene, normalized by *Z*-score. As described in the original study, hypocotyls of 7-d-old Arabidopsis seedlings were cut, followed by 7 d of CIM treatment and 24 d of SIM treatment. Data were collected at 3 stages of regeneration: CIM7, SIM3, and SIM8. The information was retrieved from the Beijing Institute of Genomics Data Center (accession number PRJCA005872; Wu et al. 2022).

**Figure 2.**
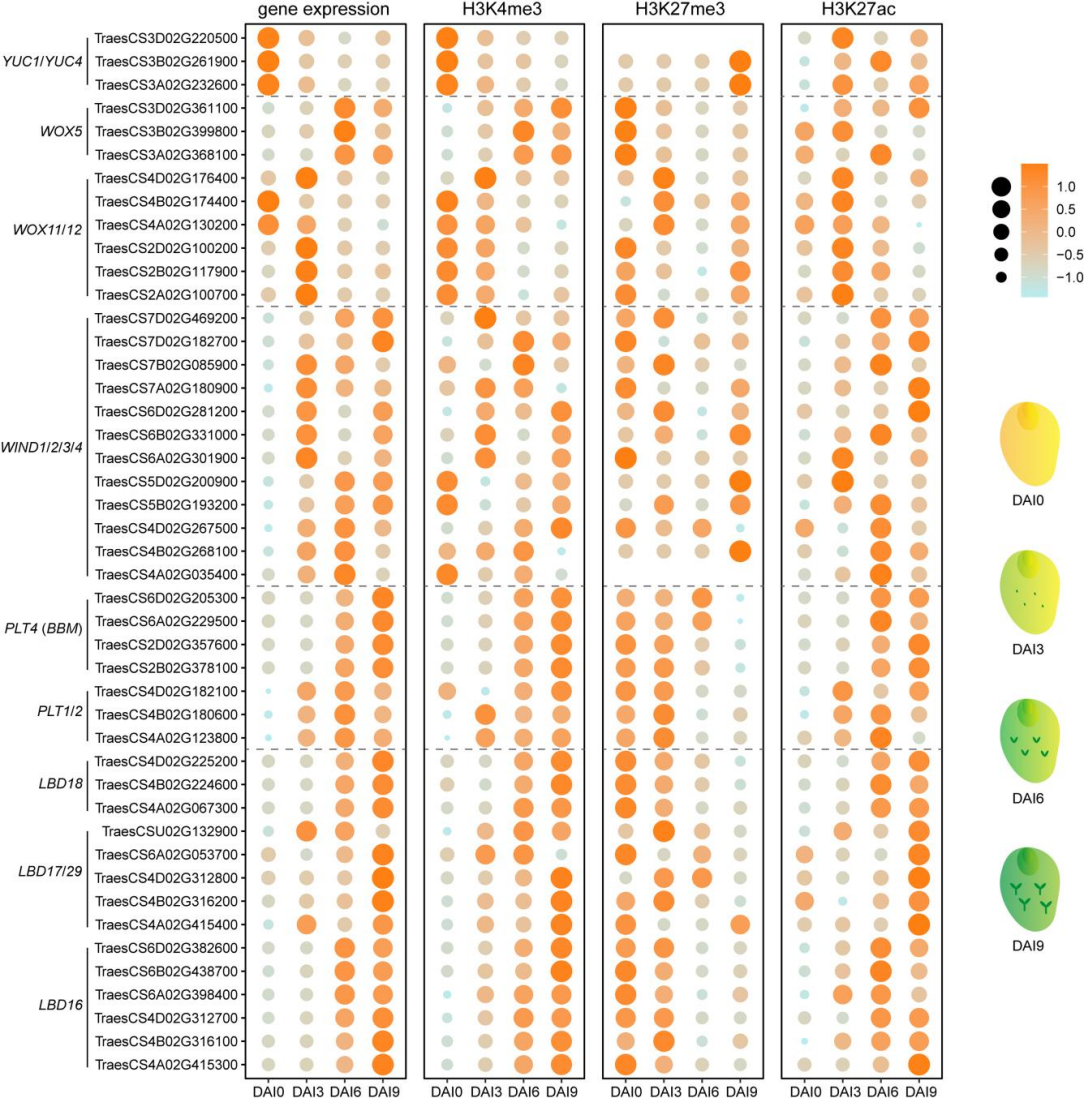
Dot plots display gene expression levels and the density of H3K4me3, H3K27me3, and H3K27ac in wheat variety Fielder at different stages of regeneration. The size of the dots represent the absolute expression level of the genes or the level of each epigenetic modification within 1.5 kb upstream and downstream of the corresponding genes, normalized by *Z*-score. The scutellum of the immature embryo, after excision of the embryo axis without tissue culture, served as the control. Wheat materials were sampled from the surface of the callus at 3, 6, and 9 d after induction (DAI). The analysis followed the procedures described in Fig. 1. Data were downloaded from the National Genomics Data Center, China National Center for Bioinformation/Beijing Institute of Genomics, Chinese Academy of Sciences (GSA: CRA008502 and CRA010204; Liu et al. 2023a).

Moreover, genome-wide profiling across multiple stages of regeneration revealed a gradual decrease in H3K27me3 modification and sequential changes in the expression of many key morphogenic genes in both Arabidopsis and wheat (*Triticum aestivum*; Figs. 1 and 2; Wu et al. 2022; Liu et al. 2023a). These findings may help explain why directly placing explants on shoot induction medium (SIM) is ineffective in triggering shoot formation. Perhaps the dilution of H3K27me3 via cell divisions on CIM is required to derepress critical regeneration-related genes for the acquisition of pluripotency.

### The role of H3K27me3 in mediating environmental signaling

During the process of plant regeneration, PRC2 mediates H3K27me3 deposition in response to not only developmental signals but also environmental stimuli, such as nutrient and light conditions. The impact of carbon source type and concentration on plant regeneration efficiency has long been recognized (Yaseen et al. 2013). However, the exact underlying mechanism has remained elusive. Recent research has shed light on this matter by revealing the role of PRC2 in mediating the influence of environmental nutrient signals on plant organogenesis (Li et al. 2017; Ye et al. 2022). This mechanism involves the evolutionarily conserved target of rapamycin (TOR) kinase signaling pathway, as FIE, a key component of Arabidopsis PRC2, serves as a direct target of TOR kinase. The phosphorylation status of FIE in response to environmental sugar signals dictates the subcellular localization of FIE and the global pattern of H3K27me3 modification. Consequently, this regulatory process influences stem cell fate and cell proliferation at the shoot and root meristems (Li et al. 2017; Ye et al. 2022).

In addition to carbon nutrients, the TOR-PRC2 pathway also transmits light signals, thereby activating cell proliferation at the shoot apical meristem (Li et al. 2017). Similarly, spatial transcriptome profiling of tomato (*Solanum lycopersicum*) callus during shoot regeneration revealed increased expression of *SlTORs* and genes related to sucrose metabolism in the shoot primordium (Song et al. 2023). Alternatively, light signals may directly regulate plant regeneration via PRC2. Notably, phytochrome B interacts with the PRC2 component VERNALIZATION INSENSITIVE 3-LIKE1/VRN5 (VIL1/VRN5) in a light-dependent manner (Kim et al. 2021). This interaction plays a role in fine-tuning H3K27me3 modification and the expression of photomorphogenesis genes (Kim et al. 2021). Such a mechanism may also be involved in regulating the light responses of genes associated with plant regeneration (Nameth et al. 2013; Chen et al. 2016; Blair Nameth et al. 2018; Dai et al. 2022).

## Role of H3K4me3 in regulating plant regeneration

### Effects of H3K4me3 deposition

During plant regeneration, changes in the expression of key developmental genes, such as *LEAFY COTYLEDON 2* (*LEC2*), *BABY BOOM* (*BBM*), and *WOX5*, coincide with dynamic changes in H3K4me3 modification, pointing to the involvement of H3K4me3 histone modification in regulating the transition of cell identities during plant regeneration (Liu et al. 2023a). Notably, the rise in H3K4me3 modification across pivotal regeneration-promoting genes often coincides with a decrease in H3K27me3 modification (Zhao et al. 2020; Liu et al. 2023a). This suggests that the function of PRC2 and Trithorax (Trx) chromatin modifiers is dynamically regulated to ensure proper cell fate transition. In Arabidopsis, H3K4 methylation is catalyzed by 7 proteins that share homology with *Drosophila* Trx, including ARABIDOPSIS TRITHORAX (ATX) 1 to 5, ARABIDOPSIS TRITHORAX-RELATED 3 (ATXR3), and ATXR7 (Zhou et al. 2020). ATXR3, also known as SET DOMAIN GROUP 2 (SDG2), serves as the primary H3K4me3 methyltransferase (Guo et al. 2010). A deficiency in ATXR3 results in reduced H3K4me3 deposition at nearly half of all H3K4me3 sites in the genome, leading to pleiotropic developmental defects in Arabidopsis (Guo et al. 2010; Yao et al. 2013; Chen et al. 2017). ATX3, ATX4, and ATX5 redundantly deposit di- and trimethylation at H3K4 at sites that partially overlap with those of ATXR3 (Chen et al. 2017). ATX1 and ATX2 appear to act at a limited number of loci, and their deficiency does not seem to have major impacts on the overall accumulation of H3K4me3 (Alvarez-Venegas and Avramova 2005; Saleh et al. 2008).

Genome-wide profiling revealed that ATX4 is responsible for H3K4me3 deposition and the active expression of shoot identity genes in Arabidopsis (Lee et al. 2019a). As a result, the deficiency in ATX4 function in leaf explants led to increased callus formation but a reduced capacity for shoot regeneration (Lee et al. 2019a). This outcome corresponds with the observation that Arabidopsis *clf-50 swn-1* mutants, which are characterized by deficiencies in the silencing of leaf identity genes, fail to initiate callus formation (He et al. 2012). In addition to ATX4, other H3K4me3 methyltransferases may also contribute to the regulation of plant regeneration. For instance, ATXR3 is essential for maintaining the root stem cell niche (Yao et al. 2013).

### Effects of H3K4me3 demethylation

Two classes of histone H3K4me3 demethylases have been identified in plants: FAD-dependent lysine-specific histone demethylases (such as homologs of human Lysine-Specific Demethylase 1 [LSD1]) and JmjC domain proteins (Lu et al. 2008). The Arabidopsis genome contains 4 homologs of human *LSD1*, known as *FLOWERING LOCUS D* (*FLD*) and *LSD1-LIKE1-3* (*LDL1-3*; Jiang et al. 2007). LDL1, LDL2, and FLD share high similarities in protein sequences and domain arrangements, and they exhibit partial functional redundancy in removing H3K4me1/2 modifications at their target loci (Jiang et al. 2007; Spedaletti et al. 2008; Hung et al. 2018, 2020; Hirakawa et al. 2019; Martignago et al. 2019; Fang et al. 2020; Inagaki et al. 2021; Noh et al. 2021; Xu et al. 2023b). In contrast, LDL3 lacks sequence conservation compared to the 3 other LSD1 homologs, indicating functional divergence (Martignago et al. 2019). Notably, a deficiency in H3K4me2 demethylation in Arabidopsis *ldl3* mutants, but not in mutants of other *LDL* homologs, impairs shoot formation (Ishihara et al. 2019). Interestingly, H3K4me2 demethylation does not immediately lead to changes in gene expression but instead primes genes for responsiveness during later stages of shoot regeneration (Ishihara et al. 2019).

The Arabidopsis genome contains 21 genes encoding JmjC domain–containing proteins. Of these, JmjC14-19 share domains and structural conservation with human KDM5/JARID1 and are categorized as putative H3K4 demethylases (Lu et al. 2008). These H3K4me-specific JMJ proteins are involved in various biological processes, such as seed germination, photomorphogenesis, the floral transition, leaf senescence, stress tolerance, and so on (Lu et al. 2010; Yang et al. 2012a, 2012b; Shen et al. 2014; Ning et al. 2015; Huang et al. 2019; Liu et al. 2019; Islam et al. 2021; Wang et al. 2021). Nevertheless, their precise roles in regulating plant regeneration remain largely uncharacterized.

## Role of histone acetylation in plant regeneration

### Effects of histone acetylation

Acetylation of histone lysine residues neutralizes the positive charges of nucleosomes, reducing their interactions with negatively charged DNA, thereby enhancing the accessibility of transcription factors and the transcriptional machinery (Kumar et al. 2021). Plants contain 4 distinct families of histone acetyltransferases, including the General Control Non-depressible 5 (GCN5) family acetyltransferases (HAG), the Moz-Ybf2/Sas3-Sas2/Tip60 (MYST) family acetyltransferases (HAM), the cAMP-Responsive Element-Binding Protein-binding protein (CBP) family acetyltransferases (HAC), and the TATA-binding Protein-Associated Factor (TAFII250) family acetyltransferases (HAF; Pandey et al. 2002). Histone acetylation is typically linked with gene activation and plays a pivotal role in regulating various biological processes in plants, including the response to wounding and plant regeneration (Kim et al. 2018; Rymen et al. 2019). Notably, genes that respond to wounding and exhibit early induction are often predeposited with H3K9/14Ac modifications (Rymen et al. 2019).

Studies have demonstrated the importance of histone acetylation in plant regeneration. Both Arabidopsis and rice *hag1* mutants exhibit abnormalities in the root stem cell niche (Kornet and Scheres 2009; Zhou et al. 2017). In line with this observation, the formation of callus was compromised in Arabidopsis hypocotyl explants lacking HAG1 and HAG3 (Rymen et al. 2019). Interestingly, the Arabidopsis *hag1* mutant displayed accelerated callus development on CIM (Kim et al. 2018). The differing effects of HAG1 on callus formation suggest that HAG1-mediated histone acetylation plays distinct roles in regulating plant responses to wounding and plant hormones, which might be orchestrated by different downstream target genes (Kim et al. 2018). Alternatively, these varying effects might be related to the different types of assays used and tissues examined. While 1 study focused on spontaneous callus formation at the wounding site in hypocotyls (Rymen et al. 2019), the other study analyzed callus formation from root explants placed on CIM (Kim et al. 2018). It is also conceivable that cell proliferation and pluripotency are differentially influenced by HAG1. In support of this notion, even though the *hag1* mutant showed accelerated callus formation on CIM, this led to severe deficiencies in shoot regeneration, indicating that enhanced cell proliferation did not result in the efficient acquisition of cell pluripotency (Kim et al. 2018). Meanwhile, mutant plants deficient in HAG2, HAM1, and HAM3 did not exhibit substantially impaired plant regeneration (Rymen et al. 2019), which could be partially attributed to the potential functional redundancy of these histone acetyltransferases. To address this matter, experiments using chemical inhibitors targeting different histone acetyltransferase families suggested that the GNAT-MYST family, but not CBP family histone acetyltransferases, functions in wounding-induced callus formation (Rymen et al. 2019).

### The role of histone deacetylation

The Arabidopsis genome encodes 18 histone deacetylases (HDACs) categorized into 3 subfamilies: the Reduced Potassium Dependency 3/Histone Deacetylase 1 (RPD3/HDA1) family, the Silent Information Regulator 2 (SIR2) family, and the plant-specific Histone Deacetylase 2 (HD2) family (Pandey et al. 2002). Several studies have indicated that callus formation is compromised in Arabidopsis mutants such as *hda9* and *hdt1*, as well as rice *Oshda710* (*HDA1* homolog) mutants (Lee et al. 2016; Zhang et al. 2020). A different investigation evaluated de novo shoot formation in a group of Arabidopsis *HDAC* mutants, including *hda6*, *hda9*, *hda10*, *hda14*, *hda17*, *hda18*, *hda19*, and *hd2b* (Temman et al. 2023). Among these mutants, only *hda19* exhibited reduced shoot formation, and further analysis revealed that hyperacetylation and overexpression of *ENHANCER OF SHOOT REGENERATION 1* (*ESR1*) and *CUC2* were responsible for this effect in the *hda19* mutant (Temman et al. 2023). Interestingly, HDA19 was found to negatively regulate somatic embryogenesis (Morończyk et al. 2022). In this case, the observed phenotypic defects in *hda19* mutant plants were likely linked to the dysregulation of embryogenesis-related transcription factors, including LEC1, LEC2, and BBM (Morończyk et al. 2022). These findings underscore the complex and distinct roles of various HDAC proteins in plant regeneration.

### The effects of HDAC inhibitors

In addition to genetic studies, the application of chemical inhibitors targeting HDACs has also contributed to our understanding of the role of histone acetylation in regulating plant regeneration. Among these inhibitors, Trichostatin A (TSA) is one of the most commonly used. TSA treatment induces somatic embryogenesis by upregulating auxin biosynthesis (Wójcikowska et al. 2018), a phenomenon also observed in the *hda19* mutant (Morończyk et al. 2022). Furthermore, TSA treatment affects the cytokinin response (Furuta et al. 2011), leading to inhibited auxin-induced root primordium formation from hypocotyl segments and redirecting their development toward callus formation (Furuta et al. 2011). However, in other instances, TSA had inhibitory effects on callus formation in Arabidopsis leaf explants and mature rice embryos (Lee et al. 2016; Zhang et al. 2020).

Several factors may explain the contrasting effects of TSA treatment on callus formation. As discussed earlier for PRC2, the routes of the cell fate transition may depend on the types of cells and explants involved, which can be differentially affected by TSA treatment. In support of this idea, while TSA impaired callus formation in mature rice embryos, it did not influence callus formation when using rice roots and shoots as explants (Zhang et al. 2020). Furthermore, concentration-dependent effects of TSA on plant regeneration have been observed in rice and wheat (Bie et al. 2020; Zhang et al. 2020). While 0.5 *μ*M TSA treatment promoted embryonic callus formation and shoot induction in wheat, increasing the concentration to 2.5 *μ*M had an inhibitory effect (Bie et al. 2020). A similar concentration-dependent effect on plant regeneration has also been noted for nicotinamide, another HDAC inhibitor (Wang et al. 2023). In addition, TSA treatment is known to alter endogenous phytohormone production and perception (Furuta et al. 2011; Wójcikowska et al. 2018). Consequently, variations in the levels of endogenous phytohormones resulting from TSA treatment may lead to differential responses of explants to exogenously applied phytohormones.

## Epigenetic regulation of key morphogenic genes

**Figure 3.**
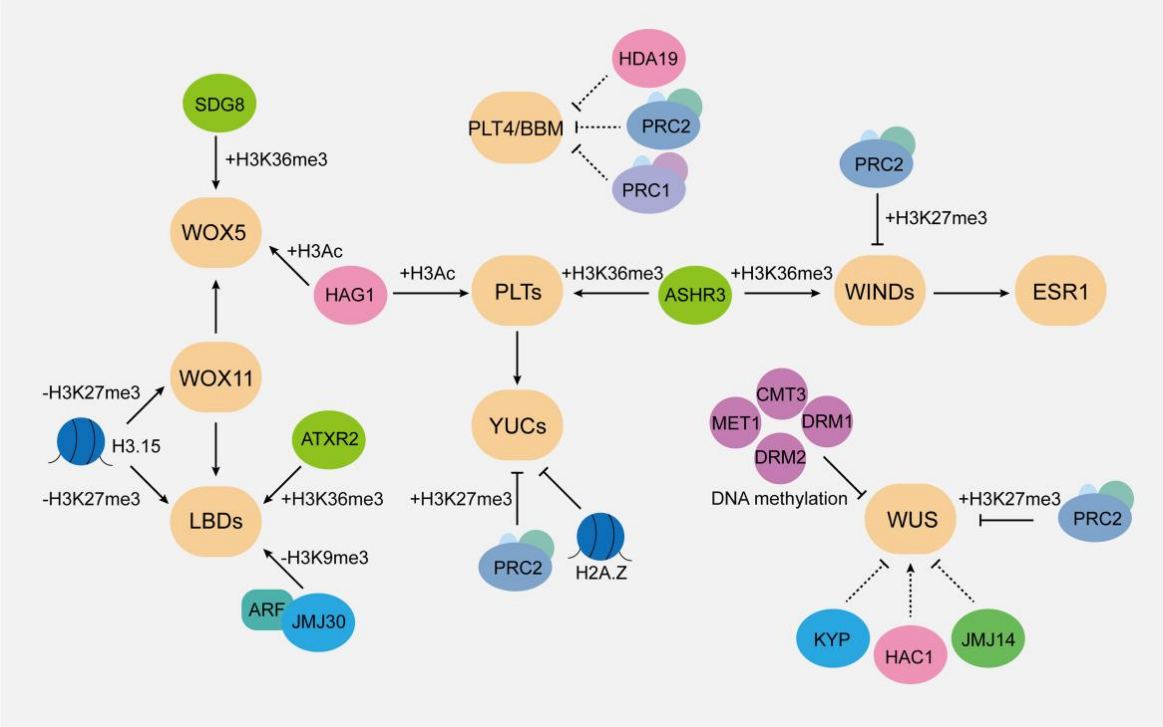
A schematic diagram of the key genes and epigenetic regulators involved in plant regeneration. Orange rounded rectangles indicate major morphogenic genes that regulate different types and stages of regeneration. Ellipses represent various types of epigenetic modifiers, each color signifying a specific type of epigenetic modification. Lines with arrowheads indicate positive regulation, while lines with bars indicate negative regulation. The solid and dotted lines represent direct and indirect regulation, respectively.

Plant regeneration is governed by a complex gene network that encompasses multiple morphogenic transcription factors. For instance, during the initial phase of organogenesis on CIM, auxin signals stimulate callus formation via the AUXIN RESPONSE FACTOR (ARF)-mediated activation of *LBDs* (Fan et al. 2012). Auxin also promotes the establishment of cellular pluripotency through 2 parallel pathways: 1 involving *WOX11* and *LBD16* (Liu et al. 2018b) and the other mediated by *PLTs* and *CUC2* (Kareem et al. 2015). Following the establishment of cell pluripotency, cytokinin-induced shoot formation on SIM is facilitated by the ARABIDOPSIS RESPONSE REGULATOR (ARR)-mediated activation of *WUS* expression (Meng et al. 2017). Wounding-induced shoot regeneration is dependent on WIND1, which activates *ESR1* expression (Iwase et al. 2017). During somatic embryogenesis, a positive feedback network has been proposed, which involves multiple morphogenic regulators, including BBM, AGAMOUS-LIKE15 (AGL15), LEC1, and LEC2 (Zheng et al. 2009; Horstman et al. 2017). These embryonic regulators stimulate the expression of *YUCCAs* (*YUCs*; Stone et al. 2008) and *INDOLEACETIC ACID-INDUCED PROTEIN30* (*IAA30*; Zheng et al. 2009) to modulate auxin biosynthesis and signaling. For a more comprehensive summary of the gene network underlying plant regeneration, we recommend excellent reviews from other authors (Ikeuchi et al. 2019; Ince and Sugimoto 2023; Liu et al. 2023b). We have chosen several key morphogenic gene families as examples to elucidate potential modes and patterns of epigenetic regulation observed during plant regeneration (Fig. 3), which we describe below.

### WUS

WUS is a homeodomain transcription factor crucial for various types of plant regeneration, including somatic embryogenesis, mesophyll regeneration, and de novo shoot organogenesis (Jha et al. 2020; Xu et al. 2021). During plant regeneration, the expression of *WUS* undergoes intricate epigenetic regulation involving both DNA methylation and histone modification. In Arabidopsis root explants, the promoter sequences of *WUS* are methylated in both the CG and CHG contexts (Li et al. 2011; Shemer et al. 2015). Incubating root explants on CIM triggers DNA demethylation, which enhances *WUS* activation on SIM (Li et al. 2011; Shemer et al. 2015). The *met1* and *drm1 drm2 cmt3* mutants show the loss or reduction of DNA methylation in the *WUS* promoter, enhancing *WUS* expression and shoot formation (Shemer et al. 2015; Liu et al. 2018a). Upon shoot induction, the expression of *WUS* is activated via 2 consecutive steps: first, the repressive histone mark H3K27me3 is removed from *WUS*; second, *WUS* is activated through the binding of the B-type ARR-HD-ZIP III complex (Zhang et al. 2017). Intriguingly, B-type ARR1 also interacts with ATXR2 and temporally promotes the expression of A-type *ARR5/7* via H3K36me3 deposition, which represses cytokinin signaling and *WUS* expression prior to shoot formation to ensure a balanced cell fate transition (Lee et al. 2021). Extensive epigenetic reprogramming involving other histone modifications was also shown to regulate the expression of *WUS*. Mutations of the H3K9 methyltransferase gene *KRYPTONITE* (*KYP*) and the H3K4 demethylase gene *JMJ14* greatly enhance *WUS* transcription, while mutations of the histone acetyltransferase gene *HAC1* reduce *WUS* transcription (Li et al. 2011).

### *WOX* genes

The WOX proteins constitute a group of plant-specific homeodomain transcription factors. Several members of this family have been demonstrated to play essential roles in various biological processes related to plant organogenesis, such as meristem maintenance, cell proliferation, and the regulation of differentiation (Tvorogova et al. 2021; Wan et al. 2023).

In rice, OsWOX11 recruits the ADA2-GCN5 histone acetyltransferase complex and the H3K27me3 demethylase JMJ705 to activate downstream target genes associated with root and shoot development (Zhou et al. 2017; Cheng et al. 2018). In Arabidopsis, the downstream targets of WOX11 and WOX12 include *LBD16* and *LBD29* (Liu et al. 2014). As ATXR2 also targets *LBD16* and *LBD29* for trimethylation at lysine 36 of histone H3 (H3K36me3) deposition to promote their expression during callus formation (Lee et al. 2017, 2018b), it has been suggested that WOX11/12 may form a complex with ATXR2 for the transcriptional activation of *LBD* genes (Lee et al. 2018a). Conversely, the expression of *WOX11* is itself epigenetically regulated. During callus formation, the deposition of the histone 3 variant H3.15 at the *WOX11* locus facilitates the removal of H3K27me1/3 and leads to the transcriptional derepression of *WOX11* (Yan et al. 2020).

In addition to the activation of *LBD16* and *LBD29*, the expression *WOX5* is also upregulated by WOX11/2 via the direct binding of WOX11/12 to its promoter (Liu et al. 2014; Hu and Xu 2016). This transcriptional activation of *WOX5* is required for callus to acquire the competence needed for de novo shoot regeneration (Kim et al. 2018). During callus formation, the expression of *WOX5* is epigenetically induced by SDG8 through H3K36me3 modification (Ma et al. 2022) and by HAG1 through histone H3 acetylation (H3Ac) modification (Kim et al. 2018). Similarly, the expression of *SHORT-ROOT* (*SHR*) and *SCARECROW* (*SCR*), encoding upstream positive regulators of *WOX5*, is also regulated by histone acetylation mediated by HAG1 and elongator complex subunit 2 (ELP2; Jia et al. 2015; Kim et al. 2018). As expected, the shoot regeneration defect of the *hag1* mutant was successfully rescued by the ectopic activation of *WOX5* and *SCR* (Kim et al. 2018). The expression of *WOX5* is also responsible for specifying the stem cell niche during root development, which involves developmental regulation via H3K27me3 and H3K4me3 modifications (Takatsuka and Umeda 2015; Zhai et al. 2020).

### PLTs

The PLT transcription factors are members of the AINTEGUMENTA-LIKE (AIL) APETALA2/ETHYLENE RESPONSIVE FACTOR (AP2/ERF), many of which are known for their roles in regulating stem cell identity in embryonic and meristem tissues (Galinha et al. 2007; Horstman et al. 2017; Xu et al. 2023a). *PLT* genes are expressed in actively dividing tissues and contribute to various regenerative responses (Santuari et al. 2016; Ikeuchi et al. 2019). Overexpressing Arabidopsis *PLT* genes induced pluripotency and stimulated cell proliferation in somatic tissues (Nole-Wilson et al. 2005; Galinha et al. 2007; Tsuwamoto et al. 2010; Krizek and Eaddy 2012).

Several *PLT* family genes were shown to be epigenetically regulated. The PRC1 homolog BMI1A/B proteins are responsible for mediating H2A monoubiquitination in Arabidopsis. This mark, in conjunction with PRC2-mediated H3K27 trimethylation, has been demonstrated to repress the expression of *BBM* (*PLT4*; Bratzel et al. 2010; Mozgová et al. 2017). In addition, the misregulated expression of *BBM* was observed in Arabidopsis explants lacking the histone deacetylase HD-TUINS PROTEIN 1 (HDT1), HDT4, and HDA19 during callus formation and somatic embryogenesis (Lee et al. 2016; Morończyk et al. 2022). Similarly, during the regeneration of *Coffea canephora*, the transition of explants from proembryogenic masses to globular embryos was found to be epigenetically regulated by DNA methylation and H3K27me3 histone modification of *BBM* (Nic-Can et al. 2013). Treatment with the DNA methylation inhibitor 5-azacytidine inhibited somatic embryogenesis by reducing the expression of *BBM* (Nic-Can et al. 2013). Furthermore, the level of DNA methylation at the *BBM* locus was influenced by treatment with phytohormones, such as 2,4-D, in a dosage-dependent manner (Grzybkowska et al. 2020).

Regarding other *PLT* genes, H3Ac at the Arabidopsis *PLT1* and *PLT2* genes is regulated by HAG1. Explants deficient in HAG1, regardless of their organ source, exhibited much reduced expression of *PLT1* and *PLT2*, along with impaired competency for shoot formation (Kim et al. 2018). Consistent with this finding, the acetylation and transcriptional activation of *PLTs* are essential for plant regeneration from Arabidopsis leaf mesophyll protoplasts by activating auxin biosynthesis genes, including *YUC1* (Sakamoto et al. 2022). Apart from histone acetylation, other epigenetic factors might also contribute to the regulation of *PLT* expression, including changes in chromatin accessibility and H3K27me3 modification (Nakamura et al. 2020; Wang et al. 2020a).

### *LBD* genes

LBD proteins constitute a family of plant-specific transcription factors distinguished by their highly conserved N-terminal LATERAL ORGAN BOUNDARIES (LOB) domain (Husbands et al. 2007; Majer and Hochholdinger 2011). The functions of LBDs include defining lateral organ boundaries, xylem differentiation, secondary growth in woody plants, and so on (Uchida et al. 2007; Soyano et al. 2008; Yordanov et al. 2010).

LBD proteins, such as LBD16, LBD17, LBD18, and LBD29, operate downstream of auxin signaling and serve as crucial promoters of callus formation (Fan et al. 2012; Xu et al. 2018). In response to the auxin signal, the expression of *LBDs* is directly activated by the sequential action of the ARF-JMJ30 complex, which mediates H3K9me3 removal, and ATXR2, which mediates H3K36me3 deposition (Lee et al. 2017, 2018b). Similar to the mechanism observed for WOX11, the induction of expression and incorporation of the histone 3 variant H3.15 upon wounding led to a reduction in H3K27me3 deposition and enhanced transcription of *LBD16*, *LBD18*, and *LBD29*, promoting callus formation (Yan et al. 2020). A comparable mechanism involving *LBD* activation via reduced H3K27me3 deposition has also been reported in peach during the leaf-to-callus transition (Zheng et al. 2022). A recent study revealed that strong expression of *TaLBD17* in wheat is associated with increased chromatin accessibility, the removal of H3K27me3 modification, and the increase in H3K4me3 modification during the late stage of callus formation from immature embryos (Liu et al. 2023a).

### WINDs

WIND1 and its homologs are AP2/ERF transcription factors that promote cell dedifferentiation and proliferation in response to wounding (Iwase et al. 2011a). The ectopic expression of *WINDs* can induce spontaneous callus formation even in the absence of exogenous phytohormones (Iwase et al. 2011a, 2011b).

Damage triggers the reprogramming of differentiated cells into stem cells (Gu et al. 2020). The rapid induction of *WIND1* following wounding was found to be orchestrated by the high levels of H3K9/14ac and H3K4me3 that were predeposited at the *WIND1* locus prior to tissue damage (Rymen et al. 2019). Further investigation revealed that the GNAT-MYST family of histone acetyltransferases, such as HAG1/GCN5 and HAG3, but not the CBP family of histone acetyltransferases, is involved in wounding-induced epigenetic reprogramming and the promotion of callus formation (Rymen et al. 2019). In addition to histone acetylation, the deposition of H3K27me3 by PRC2 also regulates the expression of *WIND3*. A deficiency in PRC2 function, as observed in the Arabidopsis *emf2 vrn2* mutant, results in the activation of *WIND* family genes and dedifferentiation of terminally differentiated root hair cells (Ikeuchi et al. 2015). Other epigenetic modifications, such as H3K36me3 deposited by ASH1-RELATED 3 (ASHR3), also activate the expression of *WIND3* (Lee et al. 2020).

### YUCs

YUC enzymes play crucial roles in catalyzing the conversion of indole-3-pyruvate (IPA) into indole-3-acetic acid (IAA), which represents a pivotal and rate-limiting step in the biosynthesis of this major auxin in plants (Won et al. 2011). In Arabidopsis, the *YUC* gene family encompasses 11 homologous genes, many of which are subjected to epigenetic regulation during plant regeneration (He et al. 2012; Gyula et al. 2018; Lambolez et al. 2022).

During the transformation from leaf tissue to callus, the repressive histone mark H3K27me3 at the *YUC4* locus diminishes, leading to the upregulation of *YUC4* expression (He et al. 2012). Similarly, when de novo root organogenesis occurs from detached leaf tissue, the activation of both *YUC1* and *YUC4* expression is associated with the removal of H3K27me3 (Chen et al. 2016). Besides *YUC* genes, the expression of another auxin biosynthesis-related gene, *ANTHRANILATE SYNTHASE α1* (*ASA1*), was shown to be epigenetically regulated by SDG8-mediated H3K36me3 deposition during de novo root regeneration from a detached leaf (Zhang et al. 2019).

In addition to H3K27me3, *YUC* genes can also be epigenetically regulated by H2A.Z during plant regeneration. Elevated temperatures (such as 27 °C) enhance shoot regeneration in Arabidopsis (Lambolez et al. 2022). This effect is partially due to the induction of *YUC4* expression by warmer temperatures, which is accompanied by a loss of H2A.Z modification (Lambolez et al. 2022). The loss of H2A.Z in *hta9 hta11* mutants promotes de novo shoot organogenesis and causes the dysregulated expression of *YUC1* and *YUC4* in a temperature-dependent manner (Lambolez et al. 2022).

Furthermore, the participation of *YUC* genes in diverse developmental processes is regulated by various epigenetic mechanisms, including histone acetylation, RdDM, and nucleosome remodeling (Gyula et al. 2018; Peng et al. 2018; Yamaguchi et al. 2018). However, whether similar mechanisms also participate in regulating *YUC* gene expression during plant regeneration remains a subject for further investigation.

## Conclusions and future perspectives

The impact of epigenetic changes on plant regeneration is complex and cannot be attributed to a single locus or a single type of epigenetic modification. Instead, it involves intricate gene regulatory networks with dynamic interactions and the balance among various epigenetic modifications (Figs. 1 and 2). Moreover, the vast diversity of plant species, the sources of explants, and the experimental treatments add a layer of complexity. Using the examples provided above, our aim was to illustrate scenarios in which epigenetic regulation is intricately integrated with the dynamic reprogramming of gene expression during plant regeneration. As specific genes are often regulated by multiple types of epigenetic modifications, the exploration of additional epigenetic regulatory circuits presents new avenues for future research (see OUTSTANDING QUESTIONS). Nevertheless, questions regarding the causal relationship between changes in gene expression and epigenetic modifications often lack simple, straightforward answers due to the presence of reciprocal feedback between transcription status and chromatin state.

Nonetheless, the continuous advancements in molecular technology hold the promise of enhancing our understanding of the role of epigenetic regulation in plant regeneration. Key developments on the horizon include gaining insights into cell type–specific epigenetic features and their impact on cell fate transitions using single-cell sequencing platforms. Additionally, we anticipate the development of advanced epigenetic engineering tools for precise regulation of morphogenic genes to break the genotype-dependent barriers of plant regeneration and transformation. These innovations are set to illuminate the intricate realm of plant regeneration. Simultaneously, plant regeneration will keep presenting us with a distinctive and ideal opportunity to develop a profound understanding of epigenetic regulatory mechanisms.

**Outstanding Questions**

- What constitutes the epigenetic fingerprint of cellular pluripotency? More precisely, how are various epigenetic modifications differentially deposited across loci encoding key morphogenic regulators when comparing cell types with regenerative potential to those without it?
- How are different epigenetic modifications balanced and coordinated to allow dynamic reprogramming of gene expression patterns required for cell fate transitions?
- How are environmental signals, such as phytohormones, wounding, light, and nutrients, harnessed to guide the epigenetic reprogramming required for organogenesis?

## Dive Curated Terms

The following phenotypic, genotypic, and functional terms are of significance to the work described in this paper:

HAG1 Gramene: AT3G54610
HAG1 Araport: AT3G54610

ATXR2 Gramene: AT3G21820
ATXR2 Araport: AT3G21820

CMT2 Gramene: AT4G19020
CMT2 Araport: AT4G19020

CMT3 Gramene: AT1G69770
CMT3 Araport: AT1G69770

LBD18 Gramene: AT2G45420
LBD18 Araport: AT2G45420

LEC1 Gramene: AT1G21970
LEC1 Araport: AT1G21970

MET1 Gramene: AT5G49160
MET1 Araport: AT5G49160

HAM1 Gramene: AT5G64610
HAM1 Araport: AT5G64610

ATX2 Gramene: AT1G05830
ATX2 Araport: AT1G05830

ATX1 Gramene: AT2G31650
ATX1 Araport: AT2G31650

YUC4 Gramene: AT5G11320
YUC4 Araport: AT5G11320

plt1 Gramene: AT3G20840
plt1 Araport: AT3G20840

PLT2 Gramene: AT1G51190
PLT2 Araport: AT1G51190

Esr1 Gramene: Zm00001d027820
Esr1 Araport: Zm00001d027820

SHR Gramene: AT4G37650
SHR Araport: AT4G37650

CYCB1;1 Gramene: AT4G37490
CYCB1;1 Araport: AT4G37490

DRM1 Gramene: AT5G15380
DRM1 Araport: AT5G15380

HAC1 Gramene: AT1G79000
HAC1 Araport: AT1G79000

JMJ12 Gramene: AT3G48430
JMJ12 Araport: AT3G48430

## References

[kiae042-B1] Abe T , Futsuhara Y. Genotypic variability for callus formation and plant regeneration in rice (*Oryza sativa* L.). Theor Appl Genet. 1986:72(1):3–10. 10.1007/BF00261446 24247763

[kiae042-B2] Aflaki F , Gutzat R, Mozgová I. Chromatin during plant regeneration: opening towards root identity? Curr Opin Plant Biol. 2022:69:102265. 10.1016/j.pbi.2022.102265 35988353

[kiae042-B3] Altpeter F , Springer NM, Bartley LE, Blechl AE, Brutnell TP, Citovsky V, Conrad LJ, Gelvin SB, Jackson DP, Kausch AP, et al Advancing crop transformation in the era of genome editing. Plant Cell. 2016:28(7):1510–1520. 10.1105/tpc.16.00196 27335450 PMC4981132

[kiae042-B4] Alvarez-Venegas R , Avramova Z. Methylation patterns of histone H3 Lys 4, Lys 9 and Lys 27 in transcriptionally active and inactive Arabidopsis genes and in atx1 mutants. Nucleic Acids Res. 2005:33(16):5199–5207. 10.1093/nar/gki830 16157865 PMC1214549

[kiae042-B5] Bairu MW , Aremu AO, Van Staden J. Somaclonal variation in plants: causes and detection methods. Plant Growth Regul. 2010:63(2):147–173. 10.1007/s10725-010-9554-x

[kiae042-B6] Becerra DC , Forero AP, Góngora GA. Age and physiological condition of donor plants affect in vitro morphogenesis in leaf explants of *Passiflora edulis* f. *flavicarpa*. Plant Cell Tissue Organ Cult. 2004:79(1):87–90. 10.1023/B:TICU.0000049440.10767.29

[kiae042-B7] Berdasco M , Alcázar R, García-Ortiz MV, Ballestar E, Fernández AF, Roldán-Arjona T, Tiburcio AF, Altabella T, Buisine N, Quesneville H, et al Promoter DNA hypermethylation and gene repression in undifferentiated *Arabidopsis* cells. PLoS One 2008:3(10):e3306. 10.1371/journal.pone.0003306 18827894 PMC2556100

[kiae042-B8] Bie XM , Dong L, Li XH, Wang H, Gao XQ, Li XG. Trichostatin A and sodium butyrate promotes plant regeneration in common wheat. Plant Signal Behav. 2020:15(12):1820681. 10.1080/15592324.2020.1820681 32962515 PMC7671042

[kiae042-B9] Bieluszewski T , Xiao J, Yang Y, Wagner D. PRC2 activity, recruitment, and silencing: a comparative perspective. Trends Plant Sci. 2021:26(11):1186–1198. 10.1016/j.tplants.2021.06.006 34294542

[kiae042-B10] Blair Nameth M , Goron TL, Dinka SJ, Morris AD, English J, Lewis D, Oro R, Raizada MN. The initial hours of post-excision light are critical for adventitious root regeneration from *Arabidopsis thaliana* (L.) Heynh. cotyledon explants. In Vitro Cell Deve Biol Plant. 2018:54(3):273–290. 10.1007/s11627-017-9880-z

[kiae042-B11] Bratzel F , López-Torrejón G, Koch M, Del Pozo JC, Calonje M. Keeping cell identity in *Arabidopsis* requires PRC1 RING-finger homologs that catalyze H2A monoubiquitination. Curr Biol. 2010:20(20):1853–1859. 10.1016/j.cub.2010.09.046 20933424

[kiae042-B12] Cao X , Du Q, Guo Y, Wang Y, Jiao Y. Condensation of STM is critical for shoot meristem maintenance and salt tolerance in *Arabidopsis*. Mol Plant. 2023:16(9):1445–1459. 10.1016/j.molp.2023.09.005 37674313

[kiae042-B13] Cardoza V , Stewart CN. *Brassica* biotechnology: progress in cellular and molecular biology. In Vitro Cell Dev Biol Plant. 2004:40(6):542–551. 10.1079/IVP2004568

[kiae042-B14] Chakrabarty D , Yu KW, Paek KY. Detection of DNA methylation changes during somatic embryogenesis of Siberian ginseng (*Eleuterococcus senticosus*). Plant Sci. 2003:165(1):61–68. 10.1016/S0168-9452(03)00127-4

[kiae042-B15] Chanvivattana Y , Bishopp A, Schubert D, Stock C, Moon YH, Sung ZR, Goodrich J. Interaction of Polycomb-group proteins controlling flowering in *Arabidopsis*. Development 2004:131(21):5263–5276. 10.1242/dev.01400 15456723

[kiae042-B16] Chen J , Wang Z, Tan K, Huang W, Shi J, Li T, Hu J, Wang K, Wang C, Xin B, et al A complete telomere-to-telomere assembly of the maize genome. Nat Genet. 2023a:55(7):1221–1231. 10.1038/s41588-023-01419-6 37322109 PMC10335936

[kiae042-B17] Chen L , Tong J, Xiao L, Ruan Y, Liu J, Zeng M, Huang H, Wang JW, Xu L. YUCCA-mediated auxin biogenesis is required for cell fate transition occurring during de novo root organogenesis in *Arabidopsis*. J Exp Bot. 2016:67(14):4273–4284. 10.1093/jxb/erw213 27255928 PMC5301932

[kiae042-B18] Chen LQ , Luo JH, Cui ZH, Xue M, Wang L, Zhang XY, Pawlowski WP, He Y. ATX3, ATX4, and ATX5 encode putative H3K4 methyltransferases and are critical for plant development. Plant Physiol. 2017:174(3):1795–1806. 10.1104/pp.16.01944 28550207 PMC5490889

[kiae042-B19] Chen Y , Hung FY, Sugimoto K. Epigenomic reprogramming in plant regeneration: locate before you modify. Curr Opin Plant Biol. 2023b:75:102415. 10.1016/j.pbi.2023.102415 37437389

[kiae042-B20] Cheng S , Tan F, Lu Y, Liu X, Li T, Yuan W, Zhao Y, Zhou DX. WOX11 recruits a histone H3K27me3 demethylase to promote gene expression during shoot development in rice. Nucleic Acids Res. 2018:46(5):2356–2369. 10.1093/nar/gky017 29361035 PMC5861455

[kiae042-B21] Crevillén P . Histone demethylases as counterbalance to H3K27me3 silencing in plants. iScience 2020:23(11):101715. 10.1016/j.isci.2020.101715 33205025 PMC7649346

[kiae042-B22] Crevillén P , Yang H, Cui X, Greeff C, Trick M, Qiu Q, Cao X, Dean C. Epigenetic reprogramming that prevents transgenerational inheritance of the vernalized state. Nature 2014:515(7528):587–590. 10.1038/nature13722 25219852 PMC4247276

[kiae042-B23] Cui X , Lu F, Qiu Q, Zhou B, Gu L, Zhang S, Kang Y, Cui X, Ma X, Yao Q, et al REF6 recognizes a specific DNA sequence to demethylate H3K27me3 and regulate organ boundary formation in *Arabidopsis*. Nat Genet. 2016:48(6):694–699. 10.1038/ng.3556 27111035

[kiae042-B24] Dai X , Wang J, Wang L, Liu Z, Li Q, Cai Y, Li S, Xiang F. HY5 inhibits in vitro shoot stem cell niches initiation via directly repressing pluripotency and cytokinin pathways. Plant J. 2022:110(3):781–801. 10.1111/tpj.15703 35132706

[kiae042-B25] Daimon Y , Takabe K, Tasaka M. The CUP-SHAPED COTYLEDON genes promote adventitious shoot formation on calli. Plant Cell Physiol. 2003:44(2):113–121. 10.1093/pcp/pcg038 12610213

[kiae042-B26] Fal K , Berr A, Le Masson M, Faigenboim A, Pano E, Ishkhneli N, Moyal NL, Villette C, Tomkova D, Chabouté ME, et al Lysine 27 of histone H3.3 is a fine modulator of developmental gene expression and stands as an epigenetic checkpoint for lignin biosynthesis in *Arabidopsis*. New Phytol. 2023:238(3):1085–1100. 10.1111/nph.18666 36779574

[kiae042-B27] Fan M , Xu C, Xu K, Hu Y. LATERAL ORGAN BOUNDARIES DOMAIN transcription factors direct callus formation in *Arabidopsis* regeneration. Cell Res. 2012:22(7):1169–1180. 10.1038/cr.2012.63 22508267 PMC3391013

[kiae042-B28] Fang X , Wu Z, Raitskin O, Webb K, Voigt P, Lu T, Howard M, Dean C. The 3′ processing of antisense RNAs physically links to chromatin-based transcriptional control. Proc Natl Acad Sci U S A. 2020:117(26):15316–15321. 10.1073/pnas.2007268117 32541063 PMC7334503

[kiae042-B29] Furuta K , Kubo M, Sano K, Demura T, Fukuda H, Liu YG, Shibata D, Kakimoto T. The CKH2/PKL chromatin remodeling factor negatively regulates cytokinin responses in *Arabidopsis* calli. Plant Cell Physiol. 2011:52(4):618–628. 10.1093/pcp/pcr022 21357580

[kiae042-B30] Galinha C , Hofhuis H, Luijten M, Willemsen V, Blilou I, Heidstra R, Scheres B. PLETHORA proteins as dose-dependent master regulators of *Arabidopsis* root development. Nature 2007:449(7165):1053–1057. 10.1038/nature06206 17960244

[kiae042-B31] Gan ES , Xu Y, Wong JY, Goh JG, Sun B, Wee WY, Huang J, Ito T. Jumonji demethylases moderate precocious flowering at elevated temperature via regulation of FLC in *Arabidopsis*. Nat Commun. 2014:5(1):5098. 10.1038/ncomms6098 25267112

[kiae042-B32] Gao Y , Ran L, Kong Y, Jiang J, Sokolov V, Wang Y. Assessment of DNA methylation changes in tissue culture of *Brassica napus*. Genetika. 2014:50(11):1338–1344. 10.7868/s001667581410004x 25739287

[kiae042-B33] Goodrich J , Puangsomlee P, Martin M, Long D, Meyerowitz EM, Coupland G. A Polycomb-group gene regulates homeotic gene expression in *Arabidopsis*. Nature 1997:386(6620):44–51. 10.1038/386044a0 9052779

[kiae042-B34] Grzybkowska D , Morończyk J, Wójcikowska B, Gaj MD. Azacitidine (5-AzaC)-treatment and mutations in DNA methylase genes affect embryogenic response and expression of the genes that are involved in somatic embryogenesis in *Arabidopsis*. Plant Growth Regul. 2018:85(2):243–256. 10.1007/s10725-018-0389-1

[kiae042-B35] Grzybkowska D , Nowak K, Gaj MD. Hypermethylation of auxin-responsive motifs in the promoters of the transcription factor genes accompanies the somatic embryogenesis induction in *Arabidopsis*. Int J Mol Sci. 2020:21(18):6849. 10.3390/ijms21186849 32961931 PMC7555384

[kiae042-B36] Gu N , Tamada Y, Imai A, Palfalvi G, Kabeya Y, Shigenobu S, Ishikawa M, Angelis KJ, Chen C, Hasebe M. DNA damage triggers reprogramming of differentiated cells into stem cells in *Physcomitrella*. Nat Plants. 2020:6(9):1098–1105. 10.1038/s41477-020-0745-9 32807952

[kiae042-B37] Guo H , Fan Y, Guo H, Wu J, Yu X, Wei J, Lian X, Zhang L, Gou Z, Fan Y, et al Somatic embryogenesis critical initiation stage-specific (m) CHH hypomethylation reveals epigenetic basis underlying embryogenic redifferentiation in cotton. Plant Biotechnol J. 2020:18(8):1648–1650. 10.1111/pbi.13336 31925881 PMC7336376

[kiae042-B38] Guo L , Yu Y, Law JA, Zhang X. SET DOMAIN GROUP2 is the major histone H3 lysine [corrected] 4 trimethyltransferase in *Arabidopsis*. Proc Natl Acad Sci U S A. 2010:107(43):18557–18562. 10.1073/pnas.1010478107 20937886 PMC2972934

[kiae042-B39] Gyula P , Baksa I, Tóth T, Mohorianu I, Dalmay T, Szittya G. Ambient temperature regulates the expression of a small set of sRNAs influencing plant development through NF-YA2 and YUC2. Plant Cell Environ. 2018:41(10):2404–2417. 10.1111/pce.13355 29856891

[kiae042-B40] He C , Chen X, Huang H, Xu L. 2012. Reprogramming of H3K27me3 is critical for acquisition of pluripotency from cultured *Arabidopsis* tissues. PLoS Genet, 8(8), e1002911. 10.1371/journal.pgen.1002911 22927830 PMC3426549

[kiae042-B41] Hirakawa T , Kuwata K, Gallego ME, White CI, Nomoto M, Tada Y, Matsunaga S. LSD1-LIKE1-mediated H3K4me2 demethylation is required for homologous recombination repair. Plant Physiol. 2019:181(2):499–509. 10.1104/pp.19.00530 31366719 PMC6776857

[kiae042-B42] Holec S , Berger F. Polycomb group complexes mediate developmental transitions in plants. Plant Physiol. 2012:158(1):35–43. 10.1104/pp.111.186445 22086420 PMC3252096

[kiae042-B43] Horstman A , Li M, Heidmann I, Weemen M, Chen B, Muino JM, Angenent GC, Boutilier K. The BABY BOOM transcription factor activates the LEC1-ABI3-FUS3-LEC2 network to induce somatic embryogenesis. Plant Physiol. 2017:175(2):848–857. 10.1104/pp.17.00232 28830937 PMC5619889

[kiae042-B44] Hossain MS , Kawakatsu T, Kim KD, Zhang N, Nguyen CT, Khan SM, Batek JM, Joshi T, Schmutz J, Grimwood J, et al Divergent cytosine DNA methylation patterns in single-cell, soybean root hairs. New Phytol. 2017:214(2):808–819. 10.1111/nph.14421 28106918

[kiae042-B45] Hsu F-M , Gohain M, Allishe A, Huang Y-J, Liao J-L, Kuang L-Y, Chen P-Y. Dynamics of the methylome and transcriptome during the regeneration of rice. Epigenomes 2018:2(3):14. 10.3390/epigenomes2030014

[kiae042-B46] Hu X , Xu L. Transcription factors WOX11/12 directly activate WOX5/7 to promote root primordia initiation and organogenesis. Plant Physiol. 2016:172(4):2363–2373. 10.1104/pp.16.01067 27784768 PMC5129711

[kiae042-B47] Huang S , Zhang A, Jin JB, Zhao B, Wang TJ, Wu Y, Wang S, Liu Y, Wang J, Guo P, et al *Arabidopsis* histone H3K4 demethylase JMJ17 functions in dehydration stress response. New Phytol. 2019:223(3):1372–1387. 10.1111/nph.15874 31038749

[kiae042-B48] Hugues A , Jacobs CS, Roudier F. Mitotic inheritance of PRC2-mediated silencing: mechanistic insights and developmental perspectives. Front Plant Sci. 2020:11:262. 10.3389/fpls.2020.00262 32211012 PMC7075419

[kiae042-B49] Hung FY , Chen C, Yen MR, Hsieh JA, Li C, Shih YH, Chen FF, Chen PY, Cui Y, Wu K. The expression of long non-coding RNAs is associated with H3Ac and H3K4me2 changes regulated by the HDA6-LDL1/2 histone modification complex in *Arabidopsis*. NAR Genom Bioinform. 2020:2(3):lqaa066. 10.1093/nargab/lqaa066 33575615 PMC7671367

[kiae042-B50] Hung FY , Chen FF, Li C, Chen C, Lai YC, Chen JH, Cui Y, Wu K. The *Arabidopsis* LDL1/2-HDA6 histone modification complex is functionally associated with CCA1/LHY in regulation of circadian clock genes. Nucleic Acids Res. 2018:46(20):10669–10681. 10.1093/nar/gky749 30124938 PMC6237806

[kiae042-B51] Husbands A , Bell EM, Shuai B, Smith HM, Springer PS. LATERAL ORGAN BOUNDARIES defines a new family of DNA-binding transcription factors and can interact with specific bHLH proteins. Nucleic Acids Res. 2007:35(19):6663–6671. 10.1093/nar/gkm775 17913740 PMC2095788

[kiae042-B52] Ikeuchi M , Favero DS, Sakamoto Y, Iwase A, Coleman D, Rymen B, Sugimoto K. Molecular mechanisms of plant regeneration. Annu Rev Plant Biol. 2019:70(1):377–406. 10.1146/annurev-arplant-050718-100434 30786238

[kiae042-B53] Ikeuchi M , Iwase A, Rymen B, Harashima H, Shibata M, Ohnuma M, Breuer C, Morao AK, De Lucas M, De Veylder L, et al PRC2 represses dedifferentiation of mature somatic cells in *Arabidopsis*. Nat Plants. 2015:1(7):15089. 10.1038/nplants.2015.89 27250255

[kiae042-B54] Ikeuchi M , Iwase A, Rymen B, Lambolez A, Kojima M, Takebayashi Y, Heyman J, Watanabe S, Seo M, De Veylder L, et al Wounding triggers callus formation via dynamic hormonal and transcriptional changes. Plant Physiol. 2017:175(3):1158–1174. 10.1104/pp.17.01035 28904073 PMC5664475

[kiae042-B55] Inagaki S , Takahashi M, Takashima K, Oya S, Kakutani T. Chromatin-based mechanisms to coordinate convergent overlapping transcription. Nat Plants. 2021:7(3):295–302. 10.1038/s41477-021-00868-3 33649596

[kiae042-B56] Ince Y , Sugimoto K. Illuminating the path to shoot meristem regeneration: molecular insights into reprogramming cells into stem cells. Curr Opin Plant Biol. 2023:76:102452. 10.1016/j.pbi.2023.102452 37709567

[kiae042-B57] Ishihara H , Sugimoto K, Tarr PT, Temman H, Kadokura S, Inui Y, Sakamoto T, Sasaki T, Aida M, Suzuki T, et al Primed histone demethylation regulates shoot regenerative competency. Nat Commun. 2019:10(1):1786. 10.1038/s41467-019-09386-5 30992430 PMC6467990

[kiae042-B58] Islam MT , Wang LC, Chen IJ, Lo KL, Lo WS. Arabidopsis JMJ17 promotes cotyledon greening during de-etiolation by repressing genes involved in tetrapyrrole biosynthesis in etiolated seedlings. New Phytol. 2021:231(3):1023–1039. 10.1111/nph.17327 33666236

[kiae042-B59] Iwase A , Harashima H, Ikeuchi M, Rymen B, Ohnuma M, Komaki S, Morohashi K, Kurata T, Nakata M, Ohme-Takagi M, et al WIND1 promotes shoot regeneration through transcriptional activation of ENHANCER OF SHOOT REGENERATION1 in Arabidopsis. Plant Cell. 2017:29(1):54–69. 10.1105/tpc.16.00623 28011694 PMC5304349

[kiae042-B60] Iwase A , Mitsuda N, Koyama T, Hiratsu K, Kojima M, Arai T, Inoue Y, Seki M, Sakakibara H, Sugimoto K, et al The AP2/ERF transcription factor WIND1 controls cell dedifferentiation in *Arabidopsis*. Curr Biol. 2011a:21(6):508–514. 10.1016/j.cub.2011.02.020 21396822

[kiae042-B61] Iwase A , Ohme-Takagi M, Sugimoto K. WIND1: a key molecular switch for plant cell dedifferentiation. Plant Signal Behav. 2011b:6(12):1943–1945. 10.4161/psb.6.12.18266 22112447 PMC3337183

[kiae042-B62] Iwgsc IWGSC . Shifting the limits in wheat research and breeding using a fully annotated reference genome. Science 2018:361:6403. 10.1126/science.aar7191 30115783

[kiae042-B63] Jha P , Ochatt SJ, Kumar V. WUSCHEL: a master regulator in plant growth signaling. Plant Cell Rep. 2020:39(4):431–444. 10.1007/s00299-020-02511-5 31984435

[kiae042-B64] Jia Y , Tian H, Li H, Yu Q, Wang L, Friml J, Ding Z. The *Arabidopsis thaliana* elongator complex subunit 2 epigenetically affects root development. J Exp Bot. 2015:66(15):4631–4642. 10.1093/jxb/erv230 25998905 PMC4507768

[kiae042-B65] Jiang D , Yang W, He Y, Amasino RM. Arabidopsis relatives of the human lysine-specific demethylase1 repress the expression of FWA and FLOWERING LOCUS C and thus promote the floral transition. Plant Cell. 2007:19(10):2975–2987. 10.1105/tpc.107.052373 17921315 PMC2174716

[kiae042-B66] Kareem A , Durgaprasad K, Sugimoto K, Du Y, Pulianmackal AJ, Trivedi ZB, Abhayadev PV, Pinon V, Meyerowitz EM, Scheres B, et al PLETHORA genes control regeneration by a two-step mechanism. Curr Biol. 2015:25(8):1017–1030. 10.1016/j.cub.2015.02.022 25819565 PMC4829346

[kiae042-B67] Kawakatsu T , Stuart T, Valdes M, Breakfield N, Schmitz RJ, Nery JR, Urich MA, Han X, Lister R, Benfey PN, et al Unique cell-type-specific patterns of DNA methylation in the root meristem. Nat Plants. 2016:2(5):16058. 10.1038/nplants.2016.58 27243651 PMC4855458

[kiae042-B68] Kim J , Bordiya Y, Kathare PK, Zhao B, Zong W, Huq E, Sung S. Phytochrome B triggers light-dependent chromatin remodelling through the PRC2-associated PHD finger protein VIL1. Nat Plants. 2021:7(9):1213–1219. 10.1038/s41477-021-00986-y 34354260 PMC8448934

[kiae042-B69] Kim JY , Yang W, Forner J, Lohmann JU, Noh B, Noh YS. Epigenetic reprogramming by histone acetyltransferase HAG1/AtGCN5 is required for pluripotency acquisition in *Arabidopsis*. EMBO J. 2018:37(20):e98726. 10.15252/embj.201798726 30061313 PMC6187204

[kiae042-B70] Kinoshita T , Harada JJ, Goldberg RB, Fischer RL. Polycomb repression of flowering during early plant development. Proc Natl Acad Sci U S A. 2001:98(24):14156–14161. 10.1073/pnas.241507798 11698668 PMC61184

[kiae042-B71] Kornet N , Scheres B. Members of the GCN5 histone acetyltransferase complex regulate PLETHORA-mediated root stem cell niche maintenance and transit amplifying cell proliferation in Arabidopsis. Plant Cell. 2009:21(4):1070–1079. 10.1105/tpc.108.065300 19376933 PMC2685635

[kiae042-B72] Krizek BA , Eaddy M. AINTEGUMENTA-LIKE6 regulates cellular differentiation in flowers. Plant Mol Biol. 2012:78(3):199–209. 10.1007/s11103-011-9844-3 22076630

[kiae042-B73] Kumar V , Thakur JK, Prasad M. Histone acetylation dynamics regulating plant development and stress responses. Cell Mol Life Sci. 2021:78(10):4467–4486. 10.1007/s00018-021-03794-x 33638653 PMC11072255

[kiae042-B74] Lambolez A , Kawamura A, Takahashi T, Rymen B, Iwase A, Favero DS, Ikeuchi M, Suzuki T, Cortijo S, Jaeger KE, et al Warm temperature promotes shoot regeneration in *Arabidopsis thaliana*. Plant Cell Physiol. 2022:63(5):618–634. 10.1093/pcp/pcac017 35157760

[kiae042-B75] Law JA , Jacobsen SE. Molecular biology. Dynamic DNA methylation. Science 2009:323(5921):1568–1569. 10.1126/science.1172782 19299607

[kiae042-B76] Lee K , Park OS, Choi CY, Seo PJ. ARABIDOPSIS TRITHORAX 4 facilitates shoot identity establishment during the plant regeneration process. Plant Cell Physiol. 2019a:60(4):826–834. 10.1093/pcp/pcy248 30605532

[kiae042-B77] Lee K , Park OS, Go JY, Yu J, Han JH, Kim J, Bae S, Jung YJ, Seo PJ. Arabidopsis ATXR2 represses de novo shoot organogenesis in the transition from callus to shoot formation. Cell Rep. 2021:37(6):109980. 10.1016/j.celrep.2021.109980 34758306

[kiae042-B78] Lee K , Park OS, Jung SJ, Seo PJ. Histone deacetylation-mediated cellular dedifferentiation in *Arabidopsis*. J Plant Physiol. 2016:191:95–100. 10.1016/j.jplph.2015.12.006 26724747

[kiae042-B79] Lee K , Park O-S, Lee HG, Seo PJ. The ASHR3 SET-domain protein is a pivotal upstream coordinator for wound-induced callus formation in *Arabidopsis*. J Plant Biol. 2020:63(5):361–368. 10.1007/s12374-020-09259-1

[kiae042-B80] Lee K , Park OS, Seo PJ. Arabidopsis ATXR2 deposits H3K36me3 at the promoters of LBD genes to facilitate cellular dedifferentiation. Sci Signal. 2017:10(507):eaan0316. 10.1126/scisignal.aan0316 29184030

[kiae042-B81] Lee K , Park OS, Seo PJ. ATXR2 as a core regulator of de novo root organogenesis. Plant Signal Behav. 2018a:13(3):e1449543. 10.1080/15592324.2018.1449543 29517958 PMC5927682

[kiae042-B82] Lee K , Park OS, Seo PJ. JMJ30-mediated demethylation of H3K9me3 drives tissue identity changes to promote callus formation in *Arabidopsis*. Plant J. 2018b:95(6):961–975. 10.1111/tpj.14002 29923261

[kiae042-B83] Lee K , Seo PJ. Dynamic epigenetic changes during plant regeneration. Trends Plant Sci. 2018:23(3):235–247. 10.1016/j.tplants.2017.11.009 29338924

[kiae042-B84] Lee LR , Wengier DL, Bergmann DC. Cell-type-specific transcriptome and histone modification dynamics during cellular reprogramming in the *Arabidopsis* stomatal lineage. Proc Natl Acad Sci U S A. 2019b:116(43):21914–21924. 10.1073/pnas.1911400116 31594845 PMC6815143

[kiae042-B85] Li W , Liu H, Cheng ZJ, Su YH, Han HN, Zhang Y, Zhang XS. DNA methylation and histone modifications regulate de novo shoot regeneration in *Arabidopsis* by modulating WUSCHEL expression and auxin signaling. PLoS Genet. 2011:7(8):e1002243. 10.1371/journal.pgen.1002243 21876682 PMC3158056

[kiae042-B86] Li X , Cai W, Liu Y, Li H, Fu L, Liu Z, Xu L, Liu H, Xu T, Xiong Y. Differential TOR activation and cell proliferation in *Arabidopsis* root and shoot apexes. Proc Natl Acad Sci U S A. 2017:114(10):2765–2770. 10.1073/pnas.1618782114 28223530 PMC5347562

[kiae042-B87] Li X , Chen L, Zhang Q, Sun Y, Li Q, Yan J. BRIF-Seq: bisulfite-converted randomly integrated fragments sequencing at the single-cell level. Mol Plant. 2019:12(3):438–446. 10.1016/j.molp.2019.01.004 30639749

[kiae042-B88] Liu D , Mu Q, Li X, Xu S, Li Y, Gu T. The callus formation capacity of strawberry leaf explant is modulated by DNA methylation. Hortic Res. 2022:9:uhab073. 10.1093/hr/uhab073 35043170 PMC8947209

[kiae042-B89] Liu H , Zhang H, Dong YX, Hao YJ, Zhang XS. DNA METHYLTRANSFERASE1-mediated shoot regeneration is regulated by cytokinin-induced cell cycle in *Arabidopsis*. New Phytol. 2018a:217(1):219–232. 10.1111/nph.14814 28960381

[kiae042-B90] Liu J , Hu X, Qin P, Prasad K, Hu Y, Xu L. The WOX11-LBD16 pathway promotes pluripotency acquisition in callus cells during de novo shoot regeneration in tissue culture. Plant Cell Physiol. 2018b:59(4):734–743. 10.1093/pcp/pcy010 29361138

[kiae042-B91] Liu J , Sheng L, Xu Y, Li J, Yang Z, Huang H, Xu L. WOX11 and 12 are involved in the first-step cell fate transition during de novo root organogenesis in Arabidopsis. Plant Cell. 2014:26(3):1081–1093. 10.1105/tpc.114.122887 24642937 PMC4001370

[kiae042-B92] Liu P , Zhang S, Zhou B, Luo X, Zhou XF, Cai B, Jin YH, Niu D, Lin J, Cao X, et al The histone H3K4 demethylase JMJ16 represses leaf senescence in Arabidopsis. Plant Cell. 2019:31(2):430–443. 10.1105/tpc.18.00693 30712008 PMC6447021

[kiae042-B93] Liu X , Bie XM, Lin X, Li M, Wang H, Zhang X, Yang Y, Zhang C, Zhang XS, Xiao J. Uncovering the transcriptional regulatory network involved in boosting wheat regeneration and transformation. Nat Plants. 2023a:9(6):908–925. 10.1038/s41477-023-01406-z 37142750

[kiae042-B94] Liu X , Zhu K, Xiao J. Recent advances in understanding of the epigenetic regulation of plant regeneration. aBIOTECH2023b:4(1):31–46. 10.1007/s42994-022-00093-2 37220541 PMC10199984

[kiae042-B95] Loschiavo F , Pitto L, Giuliano G, Torti G, Nuti-Ronchi V, Marazziti D, Vergara R, Orselli S, Terzi M. DNA methylation of embryogenic carrot cell cultures and its variations as caused by mutation, differentiation, hormones and hypomethylating drugs. Theor Appl Genet. 1989:77(3):325–331. 10.1007/BF00305823 24232608

[kiae042-B96] Lu F , Cui X, Zhang S, Jenuwein T, Cao X. Arabidopsis REF6 is a histone H3 lysine 27 demethylase. Nat Genet. 2011:43(7):715–719. 10.1038/ng.854 21642989

[kiae042-B97] Lu F , Cui X, Zhang S, Liu C, Cao X. JMJ14 is an H3K4 demethylase regulating flowering time in *Arabidopsis*. Cell Res. 2010:20(3):387–390. 10.1038/cr.2010.27 20177424

[kiae042-B98] Lu F , Li G, Cui X, Liu C, Wang XJ, Cao X. Comparative analysis of JmjC domain-containing proteins reveals the potential histone demethylases in *Arabidopsis* and rice. J Integr Plant Biol. 2008:50(7):886–896. 10.1111/j.1744-7909.2008.00692.x 18713399

[kiae042-B99] Ma J , Li Q, Zhang L, Cai S, Liu Y, Lin J, Huang R, Yu Y, Wen M, Xu T. High auxin stimulates callus through SDG8-mediated histone H3K36 methylation in *Arabidopsis*. J Integr Plant Biol. 2022:64(12):2425–2437. 10.1111/jipb.13387 36250442

[kiae042-B100] Majer C , Hochholdinger F. Defining the boundaries: structure and function of LOB domain proteins. Trends Plant Sci. 2011:16(1):47–52. 10.1016/j.tplants.2010.09.009 20961800

[kiae042-B101] Marand AP , Chen Z, Gallavotti A, Schmitz RJ. A cis-regulatory atlas in maize at single-cell resolution. Cell2021a:184(11):3041–3055.e21. 10.1016/j.cell.2021.04.014 33964211

[kiae042-B102] Marand AP , Zhang X, Nelson J, Reis BD, & Schmitz PA, J R. Profiling single-cell chromatin accessibility in plants. STAR Protoc. 2021b:2(3):100737. 10.1016/j.xpro.2021.100737 34430912 PMC8365218

[kiae042-B103] Martignago D , Bernardini B, Polticelli F, Salvi D, Cona A, Angelini R, Tavladoraki P. The four FAD-dependent histone demethylases of *Arabidopsis* are differently involved in the control of flowering time. Front Plant Sci. 2019:10:669. 10.3389/fpls.2019.00669 31214214 PMC6558185

[kiae042-B104] Matzke MA , Mosher RA. RNA-directed DNA methylation: an epigenetic pathway of increasing complexity. Nat Rev Genet. 2014:15(6):394–408. 10.1038/nrg3683 24805120

[kiae042-B105] Meng WJ , Cheng ZJ, Sang YL, Zhang MM, Rong XF, Wang ZW, Tang YY, Zhang XS. Type-B ARABIDOPSIS RESPONSE REGULATORs specify the shoot stem cell niche by dual regulation of WUSCHEL. Plant Cell. 2017:29(6):1357–1372. 10.1105/tpc.16.00640 28576846 PMC5502443

[kiae042-B106] Morończyk J , Brąszewska A, Wójcikowska B, Chwiałkowska K, Nowak K, Wójcik AM, Kwaśniewski M, Gaj MD. Insights into the histone acetylation-mediated regulation of the transcription factor genes that control the embryogenic transition in the somatic cells of *Arabidopsis*. Cells 2022:11(5):863. 10.3390/cells11050863 35269485 PMC8909028

[kiae042-B107] Mozgová I , Muñoz-VIANA R, L HENNIG. PRC2 represses hormone-induced somatic embryogenesis in vegetative tissue of *Arabidopsis thaliana*. PLoS Genet. 2017:13(1):e1006562. 10.1371/journal.pgen.1006562 28095419 PMC5283764

[kiae042-B108] Naing AH , Il Park K, Chung MY, Lim KB, Kim CK. Optimization of factors affecting efficient shoot regeneration in chrysanthemum cv. Shinma. Braz J Bot. 2015:39(4):975–984. 10.1007/s40415-015-0143-0

[kiae042-B109] Nakamura M , Batista RA, Köhler C, Hennig L. Polycomb repressive complex 2-mediated histone modification H3K27me3 is associated with embryogenic potential in Norway spruce. J Exp Bot. 2020:71(20):6366–6378. 10.1093/jxb/eraa365 32894759 PMC7586741

[kiae042-B110] Nameth B , Dinka SJ, Chatfield SP, Morris A, English J, Lewis D, Oro R, Raizada MN. The shoot regeneration capacity of excised *Arabidopsis* cotyledons is established during the initial hours after injury and is modulated by a complex genetic network of light signalling. Plant Cell Environ. 2013:36(1):68–86. 10.1111/j.1365-3040.2012.02554.x 22681544

[kiae042-B111] Nic-Can GI , Lopez-Torres A, Barredo-Pool F, Wrobel K, Loyola-Vargas VM, Rojas-Herrera R, De-La-Pena C. New insights into somatic embryogenesis: leafy cotyledon1, baby boom1 and WUSCHEL-related homeobox4 are epigenetically regulated in *Coffea canephora*. PLoS One 2013:8(8):e72160. 10.1371/journal.pone.0072160 23977240 PMC3748027

[kiae042-B112] Ning YQ , Ma ZY, Huang HW, Mo H, Zhao TT, Li L, Cai T, Chen S, Ma L, He XJ. Two novel NAC transcription factors regulate gene expression and flowering time by associating with the histone demethylase JMJ14. Nucleic Acids Res. 2015:43(3):1469–1484. 10.1093/nar/gku1382 25578968 PMC4330355

[kiae042-B113] Noh SW , Seo RR, Park HJ, Jung HW. Two *Arabidopsis* homologs of human lysine-specific demethylase function in epigenetic regulation of plant defense responses. Front Plant Sci. 2021:12:688003. 10.3389/fpls.2021.688003 34194459 PMC8236864

[kiae042-B114] Nole-Wilson S , Tranby TL, Krizek BA. AINTEGUMENTA-like (AIL) genes are expressed in young tissues and may specify meristematic or division-competent states. Plant Mol Biol. 2005:57(5):613–628. 10.1007/s11103-005-0955-6 15988559

[kiae042-B115] Ong-Abdullah M , Ordway JM, Jiang N, Ooi SE, Kok SY, Sarpan N, Azimi N, Hashim AT, Ishak Z, Rosli SK, et al Loss of Karma transposon methylation underlies the mantled somaclonal variant of oil palm. Nature 2015:525(7570):533–537. 10.1038/nature15365 26352475 PMC4857894

[kiae042-B116] Ozeki Y , Davies E, Takeda J. Somatic variation during long-term subculturing of plant cells caused by insertion of a transposable element in a phenylalanine ammonia-lyase (PAL) gene. Mol Gen Genet. 1997:254(4):407–416. 10.1007/s004380050433 9180694

[kiae042-B117] Pandey R , Müller A, Napoli CA, Selinger DA, Pikaard CS, Richards EJ, Bender J, Mount DW, Jorgensen RA. Analysis of histone acetyltransferase and histone deacetylase families of *Arabidopsis thaliana* suggests functional diversification of chromatin modification among multicellular eukaryotes. Nucleic Acids Res. 2002:30(23):5036–5055. 10.1093/nar/gkf660 12466527 PMC137973

[kiae042-B118] Peng M , Li Z, Zhou N, Ma M, Jiang Y, Dong A, Shen WH, Li L. Linking PHYTOCHROME-INTERACTING FACTOR to histone modification in plant shade avoidance. Plant Physiol. 2018:176(2):1341–1351. 10.1104/pp.17.01189 29187567 PMC5813548

[kiae042-B119] Rymen B , Kawamura A, Lambolez A, Inagaki S, Takebayashi A, Iwase A, Sakamoto Y, Sako K, Favero DS, Ikeuchi M, et al Histone acetylation orchestrates wound-induced transcriptional activation and cellular reprogramming in *Arabidopsis*. Commun Biol. 2019:2(1):404. 10.1038/s42003-019-0646-5 31701032 PMC6828771

[kiae042-B120] Sakamoto Y , Kawamura A, Suzuki T, Segami S, Maeshima M, Polyn S, De Veylder L, Sugimoto K. Transcriptional activation of auxin biosynthesis drives developmental reprogramming of differentiated cells. Plant Cell. 2022:34(11):4348–4365. 10.1093/plcell/koac218 35922895 PMC9614439

[kiae042-B121] Saleh A , Alvarez-Venegas R, Yilmaz M, Le O, Hou G, Sadder M, Al-Abdallat A, Xia Y, Lu G, Ladunga I, et al The highly similar Arabidopsis homologs of trithorax ATX1 and ATX2 encode proteins with divergent biochemical functions. Plant Cell. 2008:20(3):568–579. 10.1105/tpc.107.056614 18375658 PMC2329920

[kiae042-B122] Santos D , Fevereiro P. Loss of DNA methylation affects somatic embryogenesis in Medicago truncatula. Plant Cell Tissue Organ Cult. 2002:70(2):155–161. 10.1023/A:1016369921067

[kiae042-B123] Santuari L , Sanchez-Perez GF, Luijten M, Rutjens B, Terpstra I, Berke L, Gorte M, Prasad K, Bao D, Timmermans-Hereijgers JL, et al The PLETHORA gene regulatory network guides growth and cell differentiation in Arabidopsis roots. Plant Cell. 2016:28(12):2937–2951. 10.1105/tpc.16.00656 27920338 PMC5240741

[kiae042-B124] Shang L , He W, Wang T, Yang Y, Xu Q, Zhao X, Yang L, Zhang H, Li X, Lv Y, et al A complete assembly of the rice Nipponbare reference genome. Mol Plant. 2023:16(8):1232–1236. 10.1016/j.molp.2023.08.003 37553831

[kiae042-B125] Shemer O , Landau U, Candela H, Zemach A, Eshed Williams L. Competency for shoot regeneration from *Arabidopsis* root explants is regulated by DNA methylation. Plant Sci. 2015:238:251–261. 10.1016/j.plantsci.2015.06.015 26259192

[kiae042-B126] Shen Y , Conde ESN, Audonnet L, Servet C, Wei W, Zhou DX. Over-expression of histone H3K4 demethylase gene JMJ15 enhances salt tolerance in *Arabidopsis*. Front Plant Sci. 2014:5:290. 10.3389/fpls.2014.00290 25009544 PMC4068201

[kiae042-B127] Shibukawa T , Yazawa K, Kikuchi A, Kamada H. Possible involvement of DNA methylation on expression regulation of carrot LEC1 gene in its 5′-upstream region. Gene 2009:437(1–2):22–31. 10.1016/j.gene.2009.02.011 19264116

[kiae042-B128] Shim S , Lee HG, Park OS, Shin H, Lee K, Lee H, Huh JH, Seo PJ. Dynamic changes in DNA methylation occur in TE regions and affect cell proliferation during leaf-to-callus transition in *Arabidopsis*. Epigenetics 2022:17(1):41–58. 10.1080/15592294.2021.1872927 33406971 PMC8812807

[kiae042-B129] Song X , Guo P, Xia K, Wang M, Liu Y, Chen L, Zhang J, Xu M, Liu N, Yue Z, et al Spatial transcriptomics reveals light-induced chlorenchyma cells involved in promoting shoot regeneration in tomato callus. Proc Natl Acad Sci U S A. 2023:120(38):e2310163120. 10.1073/pnas.2310163120 37703282 PMC10515167

[kiae042-B130] Soyano T , Thitamadee S, Machida Y, Chua NH. ASYMMETRIC LEAVES2-LIKE19/LATERAL ORGAN BOUNDARIES DOMAIN30 and ASL20/LBD18 regulate tracheary element differentiation in Arabidopsis. Plant Cell. 2008:20(12):3359–3373. 10.1105/tpc.108.061796 19088331 PMC2630433

[kiae042-B131] Spedaletti V , Polticelli F, Capodaglio V, Schininà ME, Stano P, Federico R, Tavladoraki P. Characterization of a lysine-specific histone demethylase from *Arabidopsis thaliana*. Biochemistry 2008:47(17):4936–4947. 10.1021/bi701969k 18393445

[kiae042-B132] Stewart-Morgan KR , Petryk N, Groth A. Chromatin replication and epigenetic cell memory. Nat Cell Biol. 2020:22(4):361–371. 10.1038/s41556-020-0487-y 32231312

[kiae042-B133] Stone SL , Braybrook SA, Paula SL, Kwong LW, Meuser J, Pelletier J, Hsieh TF, Fischer RL, Goldberg RB, Harada JJ. Arabidopsis LEAFY COTYLEDON2 induces maturation traits and auxin activity: implications for somatic embryogenesis. Proc Natl Acad Sci U S A. 2008:105(8):3151–3156. 10.1073/pnas.0712364105 18287041 PMC2268600

[kiae042-B134] Stroud H , Ding B, Simon SA, Feng S, Bellizzi M, Pellegrini M, Wang GL, Meyers BC, Jacobsen SE. Plants regenerated from tissue culture contain stable epigenome changes in rice. Elife 2013:2:e00354. 10.7554/eLife.00354 23539454 PMC3601819

[kiae042-B135] Sugimoto K , Jiao Y, Meyerowitz EM. Arabidopsis regeneration from multiple tissues occurs via a root development pathway. Dev Cell. 2010:18(3):463–471. 10.1016/j.devcel.2010.02.004 20230752

[kiae042-B136] Sun RZ , Zuo EH, Qi JF, Liu Y, Lin CT, Deng X. A role of age-dependent DNA methylation reprogramming in regulating the regeneration capacity of *Boea hygrometrica* leaves. Funct Integr Genomics. 2020:20(1):133–149. 10.1007/s10142-019-00701-3 31414312

[kiae042-B137] Takatsuka H , Umeda M. Epigenetic control of cell division and cell differentiation in the root apex. Front Plant Sci. 2015:6:1178. 10.3389/fpls.2015.01178 26734056 PMC4689806

[kiae042-B138] Tang D , Gallusci P, Lang Z. Fruit development and epigenetic modifications. New Phytol. 2020:228(3):839–844. 10.1111/nph.16724 32506476

[kiae042-B139] Tanurdzic M , Vaughn MW, Jiang H, Lee TJ, Slotkin RK, Sosinski B, Thompson WF, Doerge RW, Martienssen RA. Epigenomic consequences of immortalized plant cell suspension culture. PLoS Biol. 2008:6(12):2880–2895. 10.1371/journal.pbio.0060302 19071958 PMC2596858

[kiae042-B140] Temman H , Sakamoto T, Ueda M, Sugimoto K, Migihashi M, Yamamoto K, Tsujimoto-Inui Y, Sato H, Shibuta MK, Nishino N, et al Histone deacetylation regulates de novo shoot regeneration. PNAS Nexus 2023:2(2):pgad002. 10.1093/pnasnexus/pgad002 36845349 PMC9944245

[kiae042-B141] Tsuwamoto R , Yokoi S, Takahata Y. Arabidopsis EMBRYOMAKER encoding an AP2 domain transcription factor plays a key role in developmental change from vegetative to embryonic phase. Plant Mol Biol. 2010:73(4–5):481–492. 10.1007/s11103-010-9634-3 20405311

[kiae042-B142] Tvorogova VE , Krasnoperova EY, Potsenkovskaia EA, Kudriashov AA, Dodueva IE, Lutova LA. What does the WOX say? Review of regulators, targets, partners. Mol Biol (Mosk). 2021:55(3):362–391. 10.1134/S002689332102031X 34097673

[kiae042-B143] Uchida N , Townsley B, Chung KH, Sinha N. Regulation of SHOOT MERISTEMLESS genes via an upstream-conserved noncoding sequence coordinates leaf development. Proc Natl Acad Sci U S A. 2007:104(40):15953–15958. 10.1073/pnas.0707577104 17898165 PMC2000400

[kiae042-B144] Wan Q , Zhai N, Xie D, Liu W, Xu L. WOX11: the founder of plant organ regeneration. Cell Regen. 2023:12(1):1. 10.1186/s13619-022-00140-9 36596978 PMC9810776

[kiae042-B145] Wang FX , Shang GD, Wu LY, Xu ZG, Zhao XY, Wang JW. Chromatin accessibility dynamics and a hierarchical transcriptional regulatory network structure for plant somatic embryogenesis. Dev Cell. 2020a:54(6):742–757.e8. 10.1016/j.devcel.2020.07.003 32755547

[kiae042-B146] Wang J , Yu C, Zhang S, Ye J, Dai H, Wang H, Huang J, Cao X, Ma J, Ma H, et al Cell-type-dependent histone demethylase specificity promotes meiotic chromosome condensation in *Arabidopsis*. Nat Plants. 2020b:6(7):823–837. 10.1038/s41477-020-0697-0 32572214

[kiae042-B147] Wang TJ , Huang S, Zhang A, Guo P, Liu Y, Xu C, Cong W, Liu B, Xu ZY. JMJ17-WRKY40 and HY5-ABI5 modules regulate the expression of ABA-responsive genes in *Arabidopsis*. New Phytol. 2021:230(2):567–584. 10.1111/nph.17177 33423315

[kiae042-B148] Wang W , Huang P, Dai W, Tang H, Qiu Y, Chang Y, Han Z, Li X, Du L, Ye X, et al Application of nicotinamide to culture medium improves the efficiency of genome editing in hexaploid wheat. Int J Mol Sci. 2023:24:4416. 10.3390/ijms24054416 36901844 PMC10002385

[kiae042-B149] Wendte JM , Schmitz RJ. Specifications of targeting heterochromatin modifications in plants. Mol Plant. 2018:11(3):381–387. 10.1016/j.molp.2017.10.002 29032247

[kiae042-B150] Whitcomb SJ , Basu A, Allis CD, Bernstein E. Polycomb group proteins: an evolutionary perspective. Trends Genet. 2007:23(10):494–502. 10.1016/j.tig.2007.08.006 17825942

[kiae042-B151] Wójcikowska B , Botor M, Morończyk J, Wójcik AM, Nodzyński T, Karcz J, Gaj MD. Trichostatin A triggers an embryogenic transition in *Arabidopsis* explants via an auxin-related pathway. Front Plant Sci. 2018:9:1353. 10.3389/fpls.2018.01353 30271420 PMC6146766

[kiae042-B152] Won C , Shen X, Mashiguchi K, Zheng Z, Dai X, Cheng Y, Kasahara H, Kamiya Y, Chory J, Zhao Y. Conversion of tryptophan to indole-3-acetic acid by TRYPTOPHAN AMINOTRANSFERASES OF ARABIDOPSIS and YUCCAs in *Arabidopsis*. Proc Natl Acad Sci U S A. 2011:108(45):18518–18523. 10.1073/pnas.1108436108 22025721 PMC3215067

[kiae042-B153] Wu LY , Shang GD, Wang FX, Gao J, Wan MC, Xu ZG, Wang JW. Dynamic chromatin state profiling reveals regulatory roles of auxin and cytokinin in shoot regeneration. Dev Cell. 2022:57(4):526–542.e7. 10.1016/j.devcel.2021.12.019 35063083

[kiae042-B154] Xu C , Cao H, Zhang Q, Wang H, Xin W, Xu E, Zhang S, Yu R, Yu D, Hu Y. Control of auxin-induced callus formation by bZIP59-LBD complex in *Arabidopsis* regeneration. Nat Plants. 2018:4(2):108–115. 10.1038/s41477-017-0095-4 29358751

[kiae042-B155] Xu C , Chang P, Guo S, Yang X, Liu X, Sui B, Yu D, Xin W, Hu Y. Transcriptional activation by WRKY23 and derepression by removal of bHLH041 coordinately establish callus pluripotency in Arabidopsis regeneration. Plant Cell. 2023a:36(1):158–173. 10.1093/plcell/koad255 37804093 PMC10734573

[kiae042-B156] Xu M , Du Q, Tian C, Wang Y, Jiao Y. Stochastic gene expression drives mesophyll protoplast regeneration. Sci Adv. 2021:7(33):eabg8466. 10.1126/sciadv.abg8466 34380624 PMC8357238

[kiae042-B157] Xu M , Li X, Xie W, Lin C, Wang Q, Tao Z. ETHYLENE INSENSITIVE3/EIN3-LIKE1 modulate FLOWERING LOCUS C expression via histone demethylase interaction. Plant Physiol. 2023b:192(3):2290–2300. 10.1093/plphys/kiad131 36852894 PMC10315263

[kiae042-B158] Yamaguchi N , Huang J, Tatsumi Y, Abe M, Sugano SS, Kojima M, Takebayashi Y, Kiba T, Yokoyama R, Nishitani K, et al Chromatin-mediated feed-forward auxin biosynthesis in floral meristem determinacy. Nat Commun. 2018:9(1):5290. 10.1038/s41467-018-07763-0 30538233 PMC6289996

[kiae042-B159] Yan A , Borg M, Berger F, Chen Z. The atypical histone variant H3.15 promotes callus formation in *Arabidopsis thaliana*. Development 2020:147(11):dev184895. 10.1242/dev.184895 32439757

[kiae042-B160] Yang H , Han Z, Cao Y, Fan D, Li H, Mo H, Feng Y, Liu L, Wang Z, Yue Y, et al A companion cell-dominant and developmentally regulated H3K4 demethylase controls flowering time in *Arabidopsis* via the repression of FLC expression. PLoS Genet. 2012a:8(4):e1002664. 10.1371/journal.pgen.1002664 22536163 PMC3334889

[kiae042-B161] Yang H , Mo H, Fan D, Cao Y, Cui S, Ma L. Overexpression of a histone H3K4 demethylase, JMJ15, accelerates flowering time in *Arabidopsis*. Plant Cell Rep. 2012b:31(7):1297–1308. 10.1007/s00299-012-1249-5 22555401

[kiae042-B162] Yao X , Feng H, Yu Y, Dong A, Shen WH. SDG2-mediated h3k4 methylation is required for proper *Arabidopsis* root growth and development. PLoS One 2013:8(2):e56537. 10.1371/journal.pone.0056537 23483879 PMC3585709

[kiae042-B163] Yaseen M , Ahmad T, Sablok G, Standardi A, Hafiz IA. Review: role of carbon sources for in vitro plant growth and development. Mol Biol Rep. 2013:40(4):2837–2849. 10.1007/s11033-012-2299-z 23212616

[kiae042-B164] Ye R , Wang M, Du H, Chhajed S, Koh J, Liu KH, Shin J, Wu Y, Shi L, Xu L, et al Glucose-driven TOR-FIE-PRC2 signalling controls plant development. Nature 2022:609(7929):986–993. 10.1038/s41586-022-05171-5 36104568 PMC9530021

[kiae042-B165] Yordanov YS , Regan S, Busov V. Members of the LATERAL ORGAN BOUNDARIES DOMAIN transcription factor family are involved in the regulation of secondary growth in *Populus*. Plant Cell. 2010:22(11):3662–3677. 10.1105/tpc.110.078634 21097711 PMC3015109

[kiae042-B166] Zakrzewski F , Schmidt M, Van Lijsebettens M, Schmidt T. DNA methylation of retrotransposons, DNA transposons and genes in sugar beet (*Beta vulgaris* L.). Plant J. 2017:90(6):1156–1175. 10.1111/tpj.13526 28257158

[kiae042-B167] Zhai H , Zhang X, You Y, Lin L, Zhou W, Li C. SEUSS integrates transcriptional and epigenetic control of root stem cell organizer specification. EMBO J. 2020:39(20):e105047. 10.15252/embj.2020105047 32926464 PMC7560201

[kiae042-B168] Zhang G , Zhao F, Chen L, Pan Y, Sun L, Bao N, Zhang T, Cui CX, Qiu Z, Zhang Y, et al Jasmonate-mediated wound signalling promotes plant regeneration. Nat Plants. 2019:5(5):491–497. 10.1038/s41477-019-0408-x 31011153

[kiae042-B169] Zhang H , Guo F, Qi P, Huang Y, Xie Y, Xu L, Han N, Xu L, Bian H. OsHDA710-mediated histone deacetylation regulates callus formation of rice mature embryo. Plant Cell Physiol. 2020:61(9):1646–1660. 10.1093/pcp/pcaa086 32592489

[kiae042-B170] Zhang H , Lang Z, Zhu JK. Dynamics and function of DNA methylation in plants. Nat Rev Mol Cell Biol. 2018:19(8):489–506. 10.1038/s41580-018-0016-z 29784956

[kiae042-B171] Zhang TQ , Lian H, Zhou CM, Xu L, Jiao Y, Wang JW. A two-step model for de novo activation of WUSCHEL during plant shoot regeneration. Plant Cell. 2017:29(5):1073–1087. 10.1105/tpc.16.00863 28389585 PMC5466026

[kiae042-B172] Zhao N , Zhang K, Wang C, Yan H, Liu Y, Xu W, Su Z. Systematic analysis of differential H3K27me3 and H3K4me3 deposition in callus and seedling reveals the epigenetic regulatory mechanisms involved in callus formation in rice. Front Genet. 2020:11:766. 10.3389/fgene.2020.00766 32765593 PMC7379484

[kiae042-B173] Zheng B , Liu J, Gao A, Chen X, Gao L, Liao L, Luo B, Ogutu CO, Han Y. Epigenetic reprogramming of H3K27me3 and DNA methylation during leaf-to-callus transition in peach. Hortic Res. 2022:9:uhac132. 10.1093/hr/uhac132 35937864 PMC9350832

[kiae042-B174] Zheng S , Hu H, Ren H, Yang Z, Qiu Q, Qi W, Liu X, Chen X, Cui X, Li S, et al The *Arabidopsis* H3K27me3 demethylase JUMONJI 13 is a temperature and photoperiod dependent flowering repressor. Nat Commun. 2019:10(1):1303. 10.1038/s41467-019-09310-x 30899015 PMC6428840

[kiae042-B175] Zheng Y , Ren N, Wang H, Stromberg AJ, Perry SE. Global identification of targets of the Arabidopsis MADS domain protein AGAMOUS-Like15. Plant Cell. 2009:21(9):2563–2577. 10.1105/tpc.109.068890 19767455 PMC2768919

[kiae042-B176] Zhou H , Liu Y, Liang Y, Zhou D, Li S, Lin S, Dong H, Huang L. The function of histone lysine methylation related SET domain group proteins in plants. Protein Sci. 2020:29(5):1120–1137. 10.1002/pro.3849 32134523 PMC7184775

[kiae042-B177] Zhou S , Jiang W, Long F, Cheng S, Yang W, Zhao Y, Zhou DX. Rice homeodomain protein wOX11 recruits a histone acetyltransferase complex to establish programs of cell proliferation of crown root meristem. Plant Cell. 2017:29(5):1088–1104. 10.1105/tpc.16.00908 28487409 PMC5466029

[kiae042-B178] Zhu D , Wen Y, Yao W, Zheng H, Zhou S, Zhang Q, Qu LJ, Chen X, Wu Z. Distinct chromatin signatures in the *Arabidopsis* male gametophyte. Nat Genet. 2023:55(4):706–720. 10.1038/s41588-023-01329-7 36864100

